# Distinct Cytoplasmic and Nuclear Functions of the Stress Induced Protein DDIT3/CHOP/GADD153

**DOI:** 10.1371/journal.pone.0033208

**Published:** 2012-04-09

**Authors:** Alexandra Jauhiainen, Christer Thomsen, Linda Strömbom, Pernilla Grundevik, Carola Andersson, Anna Danielsson, Mattias K. Andersson, Olle Nerman, Linda Rörkvist, Anders Ståhlberg, Pierre Åman

**Affiliations:** 1 Department of Medical Epidemiology and Biostatistics, Karolinska Institute, Stockholm, Sweden; 2 Sahlgrenska Cancer Center, Department of Pathology, University of Gothenburg, Gothenburg, Sweden; 3 Department of Oncology, Institute of Clinical Sciences, University of Gothenburg, Sahlgrenska University Hospital, Gothenburg, Sweden; 4 Department of Mathematical Statistics, Chalmers University of Technology, Gothenburg, Sweden; 5 Department of Mathematical Statistics, University of Gothenburg, Gothenburg, Sweden; University of North Carolina at Charlotte, United States of America

## Abstract

*DDIT3*, also known as *GADD153* or *CHOP*, encodes a basic leucine zipper transcription factor of the dimer forming C/EBP family. DDIT3 is known as a key regulator of cellular stress response, but its target genes and functions are not well characterized. Here, we applied a genome wide microarray based expression analysis to identify DDIT3 target genes and functions. By analyzing cells carrying tamoxifen inducible DDIT3 expression constructs we show distinct gene expression profiles for cells with cytoplasmic and nuclear localized DDIT3. Of 175 target genes identified only 3 were regulated by DDIT3 in both cellular localizations. More than two thirds of the genes were downregulated, supporting a role for DDIT3 as a dominant negative factor that could act by either cytoplasmic or nuclear sequestration of dimer forming transcription factor partners. Functional annotation of target genes showed cell migration, proliferation and apoptosis/survival as the most affected categories. Cytoplasmic DDIT3 affected more migration associated genes, while nuclear DDIT3 regulated more cell cycle controlling genes. Cell culture experiments confirmed that cytoplasmic DDIT3 inhibited migration, while nuclear DDIT3 caused a G1 cell cycle arrest. Promoters of target genes showed no common sequence motifs, reflecting that DDIT3 forms heterodimers with several alternative transcription factors that bind to different motifs. We conclude that expression of cytoplasmic DDIT3 regulated 94 genes. Nuclear translocation of DDIT3 regulated 81 additional genes linked to functions already affected by cytoplasmic DDIT3. Characterization of DDIT3 regulated functions helps understanding its role in stress response and involvement in cancer and degenerative disorders.

## Introduction

*DDIT3* (DNA damage induced transcript 3) also known as GADD153 (G1 arrest and DNA damage 153) or CHOP (C/EBP homologous protein), encodes a key regulator of stress response. DNA damage, ER stress, hypoxia, and starvation induce *DDIT3* transcription, mRNA stability, and translation, resulting in DDIT3 protein accumulation [1], [2], [3], [4], [5], [6], [7], [8], [9]. The biological activity of the DDIT3 protein is further regulated by phosphorylation [10]. Forced expression of *DDIT3* triggers cell cycle arrest and in some cell types apoptosis, indicating a central role in these stress effects [11], [12]. *DDIT3* has been implicated in stress responses leading to death of pancreatic insulin producing β-cells and in neurodegenerative disorders [8], [13], [14] . *DDIT3* is also involved in differentiation of specialized tissues and cells [15], [16], [17], [18], [19] and as an oncogene in sarcomas [20], [21].

The *DDIT3* encoded protein is a basic leucine zipper (BZIP) transcription factor of the dimer forming C/EBP family [18], [22]. Unlike other C/EBP transcription factors, DDIT3 cannot form homodimers. Instead, DDIT3 acts as a dominant negative factor that blocks the activities of other C/EBP proteins by forming heterodimers. However, DDIT3 can also bind DNA as a heterodimer and induce transcription of down-stream target genes [23]. An intrinsically disordered domain in DDIT3 was reported to bind proteins other than leucine zippers [24]. DDIT3 was initially regarded as a nuclear protein but has also been reported to be expressed as a cytoplasmic protein in erythroid leukemia and kidney proximal tubular epithelial cells [25], [26]. In mouse fibroblasts, nuclear localization of DDIT3 was shown to depend on dimerization with the short isoform of CEBPB (also known as LIP) [27], [28]. The cytoplasmic localization indicate functions others than direct transcriptional regulation, and raise the question whether nuclear and cytoplasmic DDIT3 trigger distinct responses.

Here, we show a predominantly cytoplasmic localization of stress induced DDIT3 in human fibroblasts and sarcoma cells. The effects of cytoplasmic and nuclear DDIT3 protein were studied in stably transfected cell clones that express high levels of DDIT3 fused to the ligand binding parts of the mouse estrogen receptor (mor). The mor part retains the recombinant protein in the cytoplasm and addition of tamoxifen induces a rapid translocation of the recombinant DDIT3 from cytoplasm to the nuclei of stably transfected cells. Employing microarray, reverse transcription quantitative real-time PCR, and western blot methods, we identified DDIT3 regulated genes and functions. Our analysis shows that cytoplasmic and nuclear DDIT3 induced distinct gene expression patterns and functions.

## Materials and Methods

### Expression vectors

The full length coding region of *DDIT3* was cloned into the pEGFP-N1 vector (Clontech Laboratories, Inc.) in frame with the EGFP [29]. MorEGFP vectors were constructed by an in-frame ligation of the morLBD construct upstream of the gene encoding EGFP. All constructs were confirmed by sequencing. The mouse estrogen receptor ligand binding domain (morLBD) construct was made by mutating the wild-type mouse oestrogen receptor (a kind gift from Dr. M. Parker). The ligand binding domain (DNA encoding amino acids 290–599) of the receptor was cloned using the primer set: MORLBD BamHI-U (5′-TATGGATCCAGGAGACATGAGGGCTGCCAACCTTTG-3′) and MORLBD BamHI-L (5′-TATGGATCCATCGTGTTGGGGAAGCCCTCT-3′). The G525R point mutation was introduced by PCR mutagenesis and amplification of circular DNA in vitro [30] using the primer set: MORLBD mut-U (5′-GGCACATGAGTAACAAACGCATGG-3′) and MORLBD mut-L (5′ATGTTGTAGAGATGCTCCATGCGTTTGTT-3′). The morLBD G525R mutant is unable to bind estrogen, but retains affinity for the synthetic ligand 4-hydroxy-tamoxifen (Sigma-Aldrich). For nuclear translocation of mutant morLBD fused DDIT3-GFP in transfected cells, 4-hydroxy-tamoxifen was added to the medium at a final concentration of 100 nM [31].

### Cell cultures and RNA extraction

The GOT3 liposarcoma cell line, human F470 fibroblasts and human parental fibrosarcoma cell line HT1080 and transfected clones were cultured at 37°C and 5% CO_2_ in RPMI 1640 medium supplemented with 2 mM L-glutamine, 50 U/ml penicillin, 50 g/ml streptomycin and 8% FCS (all Invitrogen). G418 (200 g/ml, Invitrogen) was constantly added to the transfected clones to ensure stable expression of EGFP constructs in the cell population. Low fetal bovine serum concentrations (1%) were used for stress induction experiments with fibroblasts and GOT3 cells to reduce fetal bovine serum induced background expression of DDIT3. Tunicamycin and etoposide (both Sigma-Aldrich) were used at final concentrations of 2 µg/ml and 30 µM, respectively. For experiments with forskolin treatment, cells were seeded in Petri dishes (35 mm) at a density of 180 000 cells per plate. Forskolin was added in a concentration of 1.8*10^−5^ M. The experiments were performed in 3 independent biological replicates.

RNA was extracted using RNAeasy extraction kit (Qiagen) from cells before, 2 and 8 hours after addition of 4-hydroxy-tamoxifen and stored at −140°C until analysis.

### Microarray experiments and analysis

RNA samples and a common human universal reference RNA (Stratagene) were used as templates for cDNA synthesis with Cy3 and Cy5 labeled nucleotides, according to the instructions for the Pronto Plus 6 labeling kit (Corning). Equal quantities of labeled cDNA and reference cDNA were hybridized to Agilent G4112F microarrays and the arrays were scanned using an Agilent G2565CA microarray scanner (Agilent Technologies). Feature extraction was performed with Agilent's Feature Extraction 10.4 Image Analysis Software.

Data analysis was performed with the open source statistical software R using the LIMMA package available within Bioconductor [32]. A loess smoother was applied to each array to remove intensity dependent trends and the arrays were quantile normalized for comparability. All spots not corresponding to human genes were removed before further analysis. The expression levels of duplicated probes were averaged. The biological replicates were made with different clones, the replicate similarities were therefore assessed using Pearson correlations. The replicates exhibited consistent high correlations (all>0.85) and no systematic deviations were found within or between clones (data not shown). For each probe on the array, the normalized log_2_-fold change (M-value) was calculated and retained for downstream analysis. To assess the changes in gene expression induced by cytoplasmic DDIT3, the M-values were compared for the zero time point in the DDIT3morEGFP cell line with the zero time point in the cell line with the morEGFP construct alone. Similarly, the differentially expressed genes induced by nuclear DDIT3 for the 2 and 8 hours time points were compared to the zero time point of the DDIT3morEGFP cell line (creating 2 so-called contrasts). The same procedure was employed in the control cell line. In order to remove any effects induced by the morEGFP construct alone, a 1.5 fold-change or higher in either contrast were discarded before further analysis. *EGR1*, with a regulation slightly larger than 1.5 fold in the morEGFP cell line, was also included because of a very large fold-change at the 8 hours time point. Genes regulated by the morEGFP construct alone were not removed from the analysis of cytoplasmic DDIT3 induced genes. Raw and normalized microarray data has been deposited according to MIAME standards in the MIAME compliant ArrayExpress database under accession number E-MEXP-2709.

### Functional annotation and network analysis

Probes on the microarrays were ranked according to their M-values (log_2_ fold-change). The response induced by cytoplasmic DDIT3 was more pronounced than for nuclear DDIT3. Therefore, a 3-fold and a 2-fold cutoff for regulated genes were used, respectively. The functional analysis of the regulated genes was generated through the use of Ingenuity Pathways Analysis (Ingenuity® Systems). Genes that met the above described expression criteria and were associated with biological functions and/or diseases in the Ingenuity Pathways Knowledge Base were considered for the analysis. Enriched functional categories and subgroups within each category among the regulated genes were identified for all gene sets, and the 4 most significant categories representing fundamental cellular functions in each set were chosen for further study. The “cancer” category was omitted, since we do not consider cancer as a cellular functional category.

Fischer's exact test [33] was used to calculate a p-value determining the probability that each biological function and/or disease assigned to that data set was due to chance alone. To generate functional networks, the differentially expressed genes were overlaid onto a global molecular network developed from information contained in the Ingenuity Pathways Knowledge Base. Networks of these focus genes were then algorithmically generated based on their connectivity.

### Cell migration assay

Wild type and stably transfected HT1080 cells were seeded to petri dishes (35 mm) in a 1∶1 ratio with 100 000 cells per well. At 80% confluence, a scratch wound was made in the cell monolayer. In experiments with tamoxifen treatment, the drug was added at the time of scratch wound. After 24 hr of incubation the cultures were fixed in 4% formaldehyde (Sigma-Aldrich) and stained with ethidium bromide (Thermo Scientific) at a final concentration of 20 µg/ml. Wounded areas were photographed on a fluorescence microscope after 24 hours incubation and the number of ethidium bromide stained cells and EGFP stained cells were counted. Cells were also counted in several non-wounded control areas. The experiment was repeated 6 times for each of the HT1080 cell lines transfected with EGFP, DDIT3-EGFP and 10 times for cell with DDIT3morEGFP or morEGFP. For detailed description of the migration assay modeling, see Material and Methods S1. Briefly, the ratios of migration rates for all EGFP stained cell types and migration rate for wild type cells can be deduced by using Bayes theorem. A Wilcoxon test was employed to test the differences in migration rates between different EGFP stained cell lines.

### Promoter analysis

Transcription factor binding sites (TFBS) were predicted using the MATCH program [34] and a collection of 652 vertebrate positions-scoring weight matrices (PWMs) from the TRANSFAC database [35]. Promoter sequences were extracted from the TRANSPro database, where 500 base pairs upstream and 100 base pairs downstream of the predicted transcription start site were selected for each gene. Matches to a PWM were considered a hit if either the core similarity score was 1.0 or the matrix similarity score exceeded 0.95 (conservative choice). CRE sites present in the promoters of the nuclear DDIT3 regulated genes at two hours were predicted by counting hits for the following PWMs: V$CREBATF_Q6, V$CREBP1CJUN_01, V$CREBP1_01, V$CREBP1_Q2, V$CREB_01, V$CREB_02, V$CREB_Q2, V$CREB_Q2_01, V$CREB_Q3, V$CREB_Q4, V$CREB_Q4_01, V$TAXCREB_01 and V$TAXCREB_02.

The enrichment of all TFBS among the regulated genes at 2 hours was tested in 2 different ways. A hypergeometric test was used to compare the proportion of hits of TFBS among the regulated genes and the non-regulated genes. This method is standard procedure, but is highly dependent on the choice of cut-off value for regulation, and may also be sensitive to the fact that the number of regulated genes is relatively small. As a complement, a permutation test was applied to all the genes present on the arrays (i.e. not based explicitly on the regulation cut-off). Detailed description of the permutation test are given in Material and Methods S2. The permutation test works as a complement to the hypergeometric test, and ideally low p-values with both methods should indicate significant enrichment of TFBS.

### Reverse transcription quantitative real-time PCR

Reverse transcription for microarray validation was performed on approximately 1 µg total RNA using SuperScript III (Invitrogen) according to the manufacturers' instructions, using a mixture of 5 µM oligo(dT) and 5 µM random hexamers (both Invitrogen) as primers. Real-time PCR measurements were performed on a LightCycler 480 (Roche) using the iQ SYBR Green Supermix (Bio-Rad) with 400 nM of each PCR primer (TAG Copenhagen A/S). Primer sequences are available in Table S1. Formation of correctly sized PCR products was confirmed by agarose gel electrophoresis for all assays and melting curve analysis for all samples. Gene expression data was normalized against *PPIA* and *HPRT* by geometric averaging [36]. The reference genes were selected using the Human Endogenous Control Gene Panel (TATAA Biocenter) and GenEx software (MultiD Analyses).

For the Forskolin experiment QuantiTect Reverse Transcription Kit and QuantiTect SYBR Green Kit (both QIAGEN) were used for reverse transcription and real-time PCR, respectively. Data was normalized against *GAPDH*.

### Fluorescence microscopy and western blot analysis

Fluorescence microscopy and western blot analysis was preformed as previously described [37]. A Leica DMI 600B microscope with a Leica DFC 360 FX camera was used for life imaging. The recording was made during a 1 hour time span with an image taken every 20 seconds (tamoxifen at a final concentration of 100 nM was added after 5 minutes). The software used for recording was the Leica Application Suite AF. Antibodies used were rabbit anti GADD153 (DDIT3), R20 (Santa Cruz Biotechnology), GAPDH, mouse monoclonal 9484 (Abcam), Histone H1, mouse monoclonal AE-4 (Santa Cruz Biotechnology), EGFP, mouse monoclonal JL-8 (Clontech), Lamin A, mouse monoclonal 133A2 (Abcam), ATF3, mouse monoclonal M04 (Nordic Biosite), ATF4, mouse monoclonal 2B3 (Sigma-Aldrich), EGR1, mouse monoclonal S-25 (Santa Cruz Biotechnology).

### Flow cytometric analysis of cell cycle phase distribution

The distributions of G1, S, and G2 cells were analyzed at 24, 48, 78 hours after tamoxifen treatment using the FACScalibur system (BD Biosciences). Cells were detached with trypsin/EDTA solution and fixed in 70% ethanol. Isolated nuclei were stained with propidium iodide staining solution containing: 0.1% Triton X-100, 10 ug/mL propidium iodide, and 100 ug/mL DNase-free RNase A in PBS (all Sigma-Aldrich). Standardized chicken and Rainbow trout red blood cells were obtained from the FACS unit at Örebro University, Sweden, and used as internal controls for DNA amount. The ModFit LT™ software (Verity Software House) was used for automatic analysis and peak detection to identify ploidy patterns. Aneuploid G1 cells were excluded from the diploid G2/M populations.

## Results

### Cytoplasmic accumulation of DDIT3 in stressed human fibroblasts and GOT3 liposarcoma cells

**Figure 1:**
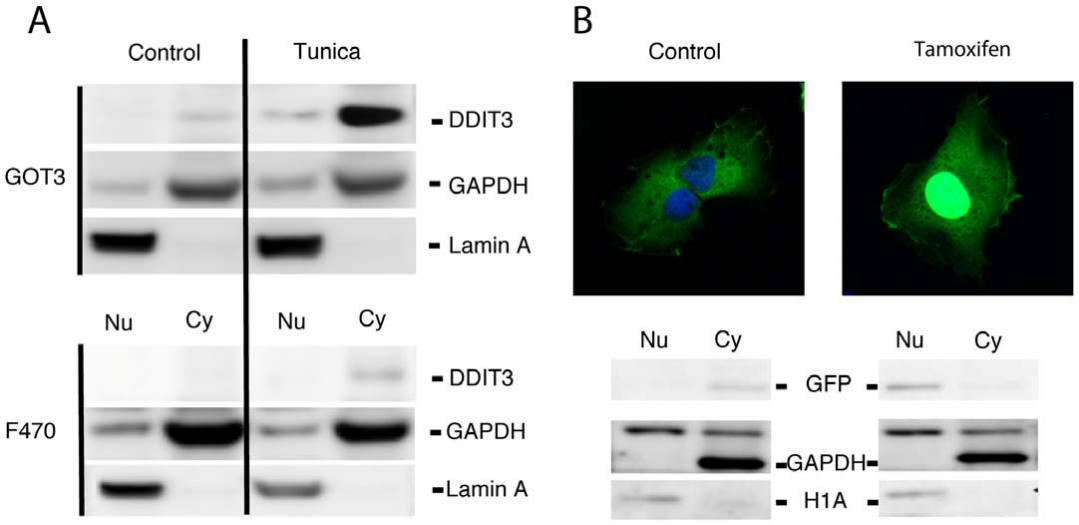
Subcellular localization of DDIT3. (A) Immunoblot analyses of nuclear (Nu) and cytoplasmic (Cy) extracts of human liposarcoma cell line GOT3 and normal human fibroblasts F470 following 8 hours of tunicamycin (2 µg/ml,Tunica) treatment. Cytoplasmic accumulation of DDIT3 is seen in both cell lines compared to untreated cells (Control). GAPDH and Lamin A are cytoplasmic and nuclear markers, respectively. (B) Confocal microscopy (upper panel) of HT1080 cells containing DDIT3morEGFP before and 1 hour after addition of tamoxifen (100 nM). A translocation of the EGFP tagged recombinant DDIT3 protein from the cytoplasm to the nucleus can be seen after the addition of tamoxifen (See also Movie S1). Immunoblot analyses (lower panel) of nuclear and cytoplasmic extracts. GAPDH and H1A were used as cytoplasmic and nuclear markers, respectively.

Native DDIT3 was first reported to be expressed as a nuclear transcription factor [18]. Here, we show that DDIT3 accumulated largely as a cytoplasmic protein in human fibroblasts and GOT3 liposarcoma cells under ER stress induced by tunicamycin interruption of protein glycosylation (Figure 1A). Similar results were obtained in human cultured fibroblasts and GOT3 liposarcoma cells treated with the DNA damaging agent etoposide (Figure S1). The GOT3 liposarcoma cell line carries a strongly amplified DDIT3 gene [21] that results in a constitutive expression of cytoplasmic DDIT3, which was further upregulated upon stress. In contrast, transfection of GOT3 cells or normal fibroblasts with a DDIT3-EGFP expression vector (not containing the mouse estrogen receptor part) resulted in a nuclear localization of the DDIT3-EGFP protein [38].

To investigate the specific effects of cytoplasmic and nuclear DDIT3 we constructed HT1080 fibrosarcoma clones with stable expression of the DDIT3morEGFP recombinant protein. Under standard culture conditions, these cells showed a cytoplasmic expression of the recombinant protein (Figure 1B and Figure S2). Tamoxifen treatment of these cells caused a nuclear translocation of the DDIT3morEGFP recombinant protein within 30 minutes (Figure 1B and Movie S1).

### Genes, functions and networks regulated downstream of cytoplasmic DDIT3morEGFP

Gene expression profiling of 41000 human transcripts was performed by microarray analysis in two *DDIT3*morEGFP transfected and two morEGFP transfected clones (For experiment overview see Figure S3). Analysis of microarray data from cells expressing cytoplasmic DDIT3morEGFP revealed 94 genes that were regulated at least 3-fold compared to their expression in morEGFP expressing cells (Table S2). Of the 94 genes, 33 genes were upregulated and 61 downregulated. To confirm the accuracy of the microarray experiments, 32 genes were selected and validated using reverse transcription quantitative real-time PCR analysis (Tables S2 and S3).

**Figure 2:**
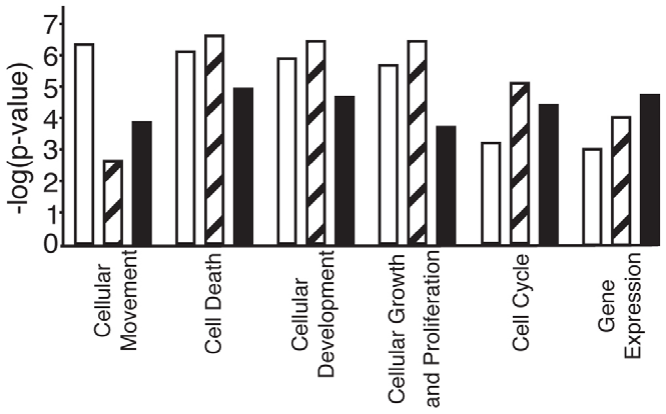
Functional gene categories regulated by cytoplasmic and nuclear DDIT3. Enrichment of functional gene categories regulated by cytoplasmic DDIT3 (white bars), and nuclear DDIT3 at 2 and 8 hours (striped bars and black bars, respectively) after tamoxifen treatment of HT1080 cells containing DDIT3morEGFP and morEGFP. The categories are ordered by the significance for cytoplasmic DDIT3 regulated genes. The y-axis shows log_10_ transformation of enrichment p-values.

Ontology analysis of identified target genes showed that the functional categories *cellular movement, cell death, cellular development,* and *cellular growth and proliferation* were the most significantly affected (Figure 2, Table S4). Some overlap between the genes annotated to the different categories was observed (Figure S4A). The 94 regulated genes were also overlayed onto the gene/protein network defined in the Ingenuity Pathway Knowledge Base with the aim to identify affected networks and network hubs (hubs are genes/proteins connecting several paths of the network). None of the DDIT3 regulated genes were found to be a major hub of the most significant networks (Figure S5).

### Cytoplasmic DDIT3 inhibits migration of HT1080 cells

Twenty of 94 regulated genes for cytoplasmic DDIT3 were annotated to the functional category *cellular movement* (Table S2). For example, *DSTN*, an actin-depolymerizing factor important for remodeling of the cytoskeleton was down regulated and *ATF3*, which has been shown to block migration, was upregulated. Several extracellular matrix related genes such as *FN1*, fibronectin, *HAS2*, hyaluronan synthetase and *CSPG4*, chondroitin sulfate proteoglycan 4, were downregulated. The adhesion molecule cadherin 11 was also downregulated. Taken together, the cytoplasmic DDIT3 regulation of movement-associated genes suggested a negative effect on cellular movement/migration.

To experimentally address the hypothesis that DDIT3 negatively regulates migration, we used a modified scratch wound migration assay. Migration was inhibited with 48% (P<0.01) for DDIT3morEGFP expressing HT1080 cells compared to morEGFP expressing cells. When tamoxifen was added to both these cell lines, the corresponding migration inhibition was 42% (P<0.01) (Material and Methods S1).

### Genes, functions and networks regulated by nuclear DDIT3morEGFP

**Figure 3:**
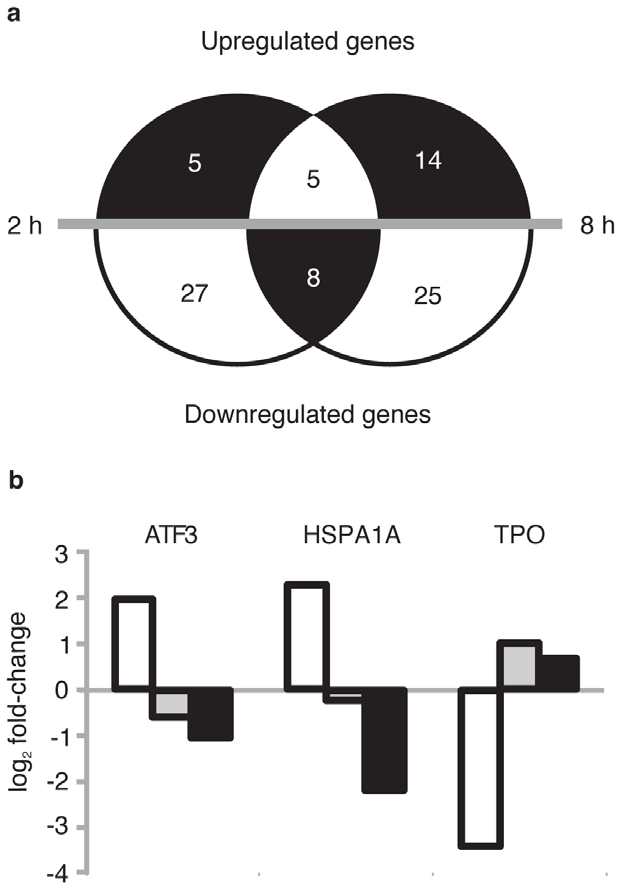
Genes regulated by cytoplasmic and nuclear DDIT3. (A) Genes regulated 2 and 8 hours after nuclear translocation of DDIT3 in DDIT3morGFP expressing HT1080 cells. (B) Genes regulated both by cytoplasmic DDIT3 and nuclear DDIT3. White bars represent regulation by cytoplasmic DDIT3. Grey and black bars represent the regulation of nuclear DDIT3 compared to levels in cells with cytoplasmic DDIT3 at 2 and 8 hours after tamoxifen addition, respectively.

To identify direct target genes and effects of nuclear DDIT3, microarray analysis was performed 2 and 8 hours after tamoxifen activation and cytoplasmic release of DDIT3 (for experiment overview see Figure S3). Only short time periods were allowed for accumulation and degradation of mRNAs regulated by nuclear DDIT3 compared to cytoplasmic DDIT3 expressing cells. Therefore, we applied a 2-fold and 3-fold cutoff for those mRNAs, respectively. After 2 hours, 45 genes were regulated (Figure 3A and Table S2). Eight hours after DDIT3 translocation, several initially regulated genes were back to their initial expression levels, but replaced by other response genes. In total 52 genes were regulated 8 hours after DDIT3 translocation, but only 13 of these genes were regulated after 2 hours (Figure 3A and Table S2). Most of the regulated genes were repressed, supporting the hypothesis that DDIT3 acts as a dominant negative factor [18].

Functional analysis among the 2 hours response genes for nuclear DDIT3 showed that the top enriched categories were *cell death*, *cellular development*, *cellular growth and proliferation* and *cell cycle* (Figure 2). Examples of a functional subgroup within respective category were: *apoptosis* (p = 1×10^−6^), *development of cells* (p = 9×10^−4^), *growth of cells* (p = 3×10^−5^) and *interphase* (p = 8×10^−6^) (Table S4B).

*Cell death*, *gene expression*, *cellular development*, and *cell cycle* were the most significant categories enriched among the 8 hours response genes of nuclear DDIT3, however all subgroups within *cellular development* contained only a small number of genes (Figure 2 and Table S4). Genes of all categories overlapped, but not to the same extent as for the categories in the 2 hours time point (Figure S4B-C). Examples of functional subgroups of the *cell death, gene expression*, and *cell cycle* categories were *apoptosis* (p = 1×10^−5^), *transcription* (p = 2×10^−5^) and *M phase of eukaryotic* cells (p = 4×10^−4^) (Table S4).

Network analysis based on the regulated genes from 2 and 8 hours after nuclear transition of DDIT3 showed that the transcription factor gene *EGR1* may work as a hub connecting several paths in the network (Figure S5). *EGR1* was the single most upregulated gene and this regulation was confirmed at protein level by western blot analysis (Figure S1),

### Nuclear translocation of DDIT3morEGFP leads to a transient G1 arrest

**Figure 4:**
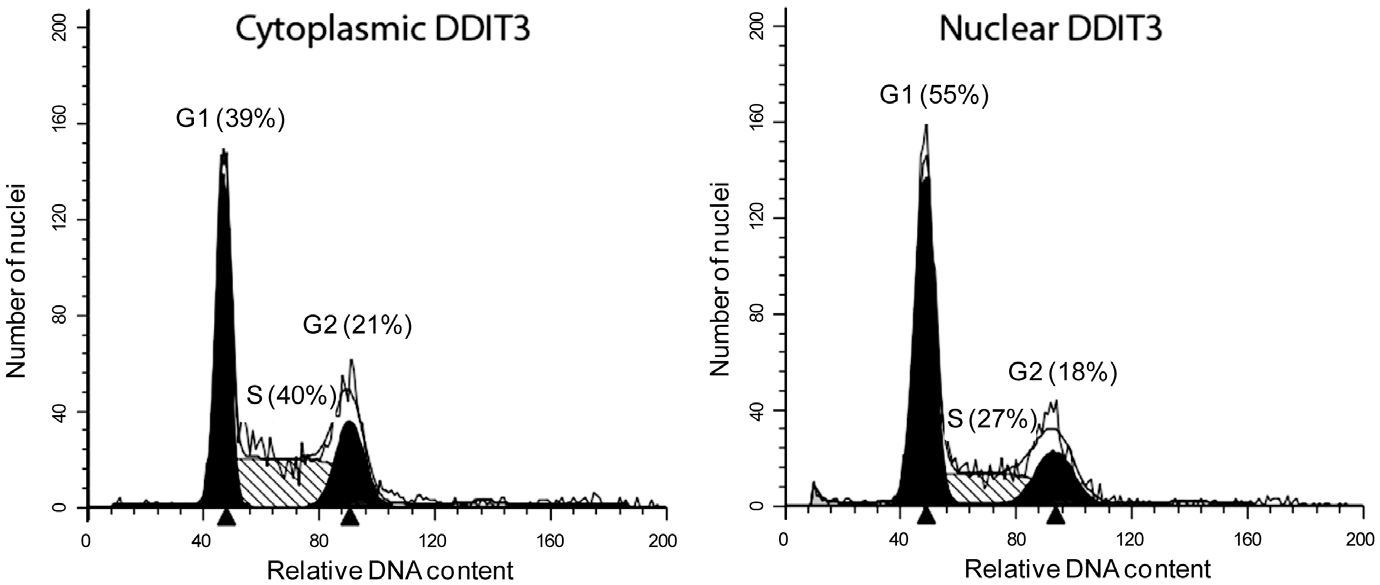
DDIT3 induced cell cycle arrest. Flow cytometry analysis of DDIT3morEGFP expressing HT1080 cells cultured with and without tamoxifen for 12 hours. Cells with nuclear DDIT3 were accumulated in G1-phase and depleted from the S- and G2-phases. Numbers of diploid cells in G1-, G2- and M-phase are shown in black and S-phase cells are shown as striped. Aneuploid cells are shown in the white overlaying histogram.

Functional analysis of our microarray results suggested that DDIT3 regulated genes were involved in cell cycle control and apoptosis. To test these functions we studied cell cycle progression and cell survival. Nuclear translocation of DDIT3morEGFP in HT1080 cells caused a transient growth arrest with accumulation of cells in the G1-phase and depletion of cells in the S- and G2-phases (Figure 4), whereas no growth effects were observed in the morEGFP expressing cells (data not shown). No increased level of apoptotic cells was observed in DDIT3morEGFP expressing cells before or after tamoxifen treatment (data not shown).

### Cytoplasmic and nuclear DDIT3 regulate different genes but with overlapping functions

Ninety-one of 94 genes regulated by cytoplasmic DDIT3 remained at their initial expression levels after the nuclear translocation of DDIT3 (Figure S6). Exceptions were *ATF3* and *HSPA1A*, which were upregulated, and *TPO*, which was downregulated in cells with cytoplasmic DDIT3 (Figure 3B). Nuclear translocation of DDIT3 partially or completely reduced the cytoplasmic DDIT3 effect on the expression of these 3 genes. ATF3 regulation was confirmed by immunoblot analysis (Figure S1). In addition to the 94 genes and functions regulated by cytoplasmic DDIT3, 81 more genes were regulated after nuclear translocation (Figure 3A, Figure S6).Target genes of nuclear DDIT3 belonged to functional categories that were already significantly enriched by cytoplasmic DDIT3 (Figure 2).

### Promoter analysis of DDIT3 regulated genes

Genes regulated by nuclear DDIT3 after 2 hours of tamoxifen treatment were considered direct targets of DDIT3. Two different scoring methods for enrichment of predicted transcription factor binding sites (TFBS) using 652 position-scoring weight matrices (PWMs) were applied. Ideally, hits to a PWM should be present in most genes regulated, but not enriched in randomly selected reference gene panels.

A few binding sites, for example V$AP2_Q6, V$SP1_Q2_01, V$CNOT3_01 and V$SRF_01, showed moderately low p-values for enrichment among the regulated genes with both scoring methods (Table S5). In addition, several of the top scoring PWMs had putative binding sites in the reference genes. Follow up analyses including clustering and functional annotation failed to identify a common pattern of binding site occurrence in the promoters of the regulated genes.

DDIT3 forms dimers with other C/EBP leucine zipper factors and acts as a dominant negative factor or binds as heterodimers to specific sites [23]. However, neither the C/EBP sites, nor the reported DDIT3 (CHOP) binding sites were enriched among the regulated genes.

Twenty of the genes regulated by nuclear DDIT3 contained a cAMP responsive element (CRE) site in their promoter region. Despite difficulties in showing significant enrichment for these CRE sites, we decided to further investigate a possible interaction with DDIT3, since several CRE binding proteins belong to the basic leucine zipper family of transcription factors that may form heterodimers with DDIT3 [39]. HT1080 cells expressing EGFP or nuclear DDIT3-EGFP were treated with the cAMP inducing agent forskolin and expression of responsive genes was analyzed by reverse transcription quantitative real-time PCR. Six genes (*DUSP1*, *FOSL2*, *JUN*, *KLF13*, *NR4A3* and *SNF1LK*) reported to be cAMP regulated were analyzed. Although the DDIT3 effects on forskolin induction varied between the genes, there was no general inhibition of the forskolin induced effect (Figure S7). This indicates that there is no general dominant negative effect of DDIT3 in cAMP regulated transcription factor complexes of these 6 genes.

## Discussion

Mammalian cells respond to stress with specific gene expression and protein activation programs, evolved to minimize damage or induce apoptosis. DDIT3 is a key regulator in stress response and DDIT3 accumulation is regulated both at transcriptional, mRNA stability and translational levels [40]. Mice lacking DDIT3 develops normally but with impaired osteoblast function [41]. However, DDIT3^−/−^ cells are resistant to ER stress mediated apoptosis [12], [42], [43] and this is reflected in animal models of cell stress associated degenerative diseases [8].

DDIT3 was initially described as a nuclear transcription factor, but later reports suggest that DDIT3 can be expressed in the cytoplasm [25], [26]. Here we show that GOT3 sarcoma cells and normal human fibroblasts accumulate cytoplasmic DDIT3 under tunicamycin and etoposide induced stress conditions. Many studies reporting nuclear localization of DDIT3 are made with cells transfected or transduced with DDIT3 expression plasmids or virus vectors. Transfection of fibroblasts and GOT3 cells with a DDIT3-EGFP expression vector also showed nuclear expression of the recombinant protein, indicating that these cells are capable of nuclear DDIT3 localization (data not shown). With these results we hypothesized that transfection induced stress could result in a nuclear DDIT3 expression. However, transient transfections of GOT3 cells with a GFP expression plasmid caused no change in the localization of endogenous DDIT3. Inspection of western blot results showed no detectable size difference between cytoplasmic and nuclear DDIT3, eliminating major protein modifications such as ubiquitinylation or sumoylation as a mechanism for the selective localization. Nuclear localization of DDIT3 in mouse fibroblasts has been reported to depend on dimerization of the short isoform, LIP, of CEBPB, which also accumulates under stress [27], [28]. CEBPB transcripts and the long (LAP) isoform of the protein are constitutively expressed in the normal human fibroblasts, the GOT3 and HT1080 cell lines used in this study [38], [44] (Figure S1). However, the LIP isoform was not detected, which may explain why these cells express DDIT3 in the cytoplasm. We conclude that ER stress and genotoxic stress caused cytoplasmic accumulation of DDIT3 in the human cell types analyzed in this study.

The observation that DDIT3 can be expressed both in the cytoplasm and the nuclei has to be accounted for when biological functions of this protein is studied. We studied the effects of cytoplasmic DDIT3 and nuclear DDIT3 by analysis of two transfected clones stably expressing recombinant DDIT3 protein fused to the ligand binding parts of a mutated mouse estrogen receptor and EGFP. The forced expression of DDIT3 differs from the normal expression pattern of DDIT3, which is restricted to stressed cells and during differentiation of some cell types. Stress responses activates, however, several signaling pathways and transcription factors that act in parallel with partly overlapping functions, making it difficult to identify direct targets of DDIT3. For example, studies of tunicamycin stressed *ddit3* ^−/−^ and wild type mouse fibroblasts, identified target genes that most likely were activated by DDIT3 in concert with other stress induced factors [45], [46], [47].

Our applied experimental system allowed us to study the cytoplasmic and nuclear effects of DDIT3 in a controlled manner. The recombinant DDIT3 was under standard conditions retained in the cytoplasmic compartment and 94 genes were down- or upregulated three times or more compared to two morEGFP control clones. Genes regulated by cytoplasmic DDIT3 were obviously not direct targets. We speculate that cytoplasmic DDIT3 could bind and sequester other BZIP transcription factors and prevent their nuclear localization. The role as a dominant negative factor is further supported by the fact that most regulated genes were suppressed, not induced (Figure 3 and Table S2). In addition to CEBPB, the cells analyzed in this study constitutively express ATF4 and low levels of ATF3 (Figure S1), thus making dimerization possible with these known partners of DDIT3 [38], [44], [48], [49]. Furthermore, the BZIP dimerization domain of DDIT3 may bind several more BZIP containing factors and thereby block/modify their activities [50]. The intrinsically disordered DDIT3 domain may also mediate binding and interactions with proteins other than leucine zippers [24]. Cytoplasmic DDIT3 may thus act through interactions both with BZIP and other proteins.

Of the 94 genes, which were regulated 3-fold or more by cytoplasmic DDIT3, only 3 (*HSP1A*, *TPO* and *ATF3*) were further regulated by nuclear DDIT3. Interestingly, these three target genes were regulated in opposite direction upon nuclear DDIT3 translocation, thus eliminating the effects of cytoplasmic DDIT3. ATF3 may form heterodimers with DDIT3 [48] and both ATF3 and DDIT3 are upregulated by stress-activated ATF4, which is another DDIT3 interacting BZIP transcription factor upstream in ER stress signaling [49], [51], [52]. In vivo and in vitro studies have shown that DDIT3 and ATF3 suppress each other's expression at the transcriptional level, leading to either DDIT3 or ATF3 expression in stressed cells and tissues. Our results, showing that cytoplasmic DDIT3 induce and nuclear DDIT3 suppress ATF3 transcription, agrees with this model and add yet another level of regulation that depends on the cellular localization of DDIT3.

In addition to the 94 genes regulated by cytoplasmic DDIT3, 84 more genes were regulated 2-fold or more by nuclear translocation of DDIT3. Moreover, these genes were in the same functional categories as the first 94 genes regulated by cytoplasmic DDIT3. This shows that nuclear translocation steps up and modulates functions already affected by cytoplasmic DDIT3. Decreasing the threshold for cytoplasmic regulated genes to a 2-fold cutoff increased the number of targets genes to 371, but only 8 of these genes were identified as target genes for nuclear DDIT3. We hypothesize that a DDIT3 leakage from the cytoplasm to the nucleus would result in common target genes, but with a difference in regulation magnitude. However, our data revealed very few overlapping genes, indicating unique properties of cytoplasmic and nuclear localized DDIT3.

Ontogeny analysis of HT1080 cells with cytoplasmic DDIT3 showed that the category *cellular movement* was significantly affected. DDIT3 regulation of several migration/movement-associated genes may provide mechanistic explanations for the impaired migration. For example, *DSTN*, which encodes an actin depolymerizing protein was downregulated in DDIT3 expressing cells. We have previously shown that DDIT3 binds cyclin dependent kinase 2 (CDK2) and that CDK2 also binds cytoskeletal proteins such as myosin 9, myosin 10 and plectin in DDIT3 expressing cells [53]. The CDK2 inhibitor CDKN1A/P21/waf1 is also downregulated by DDIT3 thus probably increasing presence of active CDK2 [54]. These DDIT3 mediated protein interactions can explain the decreased migration capacity.

Our results and ontogeny analysis suggest that nuclear DDIT3 controls cell growth. Forced expression of DDIT3 was reported to induce a G1 cell cycle arrest [11] and our experimental system recapitulated this effect when the protein was translocated to the nuclei (Figure 4). Several of the regulated genes reported here may execute the growth arrest but further investigations are needed to dissect the mechanism (Table S2).

Previous studies report DDIT3 induced apoptosis in some cell types [8]. In our experimental system no apoptosis was triggered. This could be explained by that no stress inducing agents were used and stress induced factors others than DDIT3 was not expressed. Several apoptosis controlling genes were, however, regulated and this depended on the cellular localization of DDIT3. Upon nuclear localization the apoptosis protective genes *PAX2, PHLDA1, SGK1, SPRY2*, and *SYVN1* were all downregulated, supporting a DDIT3 induction of apoptosis functions. This effect was balanced by the simultaneous downregulation of the pro-apoptotic genes *KLF6, PLK2, RND3* and *TXNIP*.

The most upregulated gene induced by nuclear DDIT3 was *EGR1* that encodes a zinc finger type transcription factor involved in a variety of biological responses and effects [55], [56], [57]. EGR1 forms a DNA binding complex with C/EBPB, which is an important dimerization partner of DDIT3 [18]. Thus, DDIT3 may act both as an inducer of EGR1 and as a transcriptional cofactor for regulation of C/EBPB-EGR1 target genes. EGR1 was also pointed out as an important node in our network analysis (Figure S5).

Screening for recurrent DDIT3 binding sites in the promoter regions of regulated genes showed no common binding motif. DDIT3 cannot form homodimers, but heterodimers with several alternative transcription factors. The heterodimers probably bind to different motifs and this may explain the lack of a common DDIT3 motif in the response genes.

In summary, we show that DDIT3 may be expressed both as a cytoplasmic and nuclear protein. Further, we show that cells expressing cytoplasmic and nuclear DDIT3 have distinct gene expression profiles, but that the target genes belong to the same functional categories. Ninety-four genes were regulated by cytoplasmic DDIT3 and 84 additional genes were regulated when DDIT3 translocated to the nuclei. Most target genes were downregulated supporting a dominant negative function of DDIT3 in transcriptional regulation. Detailed characterization of DDIT3 functions may help understanding its roles in cancer and in diseases involving cell/tissue stress.

## Supporting Information

Figure S1 **Protein data for GOT3, F470 and HT1080 cells.** (A) Etoposide induction of cytoplasmic DDIT3 in human fibroblasts and GOT3 cells. Immunoblot analysis of DDIT3 in nuclear (Nu) and cytoplasmic (Cy) extracts of human liposarcoma cell line GOT3 and normal human fibroblasts F470 following 8 hours of etoposide (30 µM, Etopos) treatment. Cytoplasmic accumulation of DDIT3 is seen in both cell lines compared to untreated cells (Control). GAPDH and Lamin A are cytoplasmic and nuclear markers, respectively. (B) Immunoblot analysis of EGR1, ATF3 and ATF4 in HT1080 cells expressing DDIT3-mor-EGFP or mor-EGFP. Addition of tamoxifen to cell cultures (8 h and 12 h) caused an up-regulation of EGR1 in cells expressing DDIT3-mor-EGFP but not in mor-EGFP control cells. The expression level of ATF3 was increased in cells expressing cytoplasmic DDIT3-mor-EGFP (0 h) as compared to control cells. Nuclear translocation of DDIT3-mor-EGFP (8 h and 12 h) caused a subsequent reduction in ATF3 levels compared to cells with cytoplasmic DDIT3-mor-EGFP. ATF4 was detected in all samples and showed no change in expression levels. GAPDH and Lamin-A were used as loading controls for EGR1 and ATF3/ATF4 respectively. (C) Immunoblot analysis of CEBPB expression in HT1080, GOT3 and cultured human fibroblast (passage 9).
(TIF)

Figure S2 **Map of the recombinant DDIT3morEGFP protein.** The protein consist of the full length DDIT3 protein juxtaposed to a mutated tamoxifen specific ligand binding part of the mouse estrogen receptor (mor) and EGFP. The mor part mediates cytoplasmic retention that is released upon addition of tamoxifen.
(TIF)

Figure S3 **Overview of design and analysis of microarray experiments.** Genes regulated by cytoplasmic DDIT3 were extracted by making the comparison indicated with (1), comparing the expression between the morEGFP and DDIT3morEGFP cell lines before addition of tamoxifen. Genes regulated by nuclear DDIT3 were extracted with the comparisons indicated by (2) and (3) within the DDIT3morEGFP cell line, for 2 and 8 hours after tamoxifen addition, respectively. Genes regulated in the morEGFP cell line were removed before analysis of nuclear DDIT3 regulation. Each square corresponds to a microarray experiment replicate.
(TIF)

Figure S4 **Overlap of genes between functional categories.** (a) Cytoplasmic DDIT3 induced genes.(b) Nuclear DDIT3 induced genes after 2 hours of tamoxifen addition. (c) Nuclear DDIT3 induced genes after 8 hours of tamoxifen addition. Four functional categories of genes are shown in (a) and the number of genes that belongs to different categories is shown with overlapping boxes. For example, one gene belongs to both Cell death and Cellular movement, while four genes belong to Cell death, Cellular development and Cellular Growth and Proliferation.
(TIFF)

Figure S5 **Interaction networks formed by the genes regulated by DDIT3.** Red hues correspond to upregulation, while green hues correspond to downregulation (no coloring means no differential expression). Vertical diamonds, horizontal diamonds, vertical ovals, and horizontal ovals represent enzymes, peptidases, transmembrane receptors, and transcription factors, respectively. Squares, rectangles, up-pointing triangles, and down-pointing triangles denote cytokines, G-protein coupled receptors, phosphatases, and kinases, in turn. Double circles, trapezoids, and single circles symbolize complexes, transporters, and finally, gene products with other functions. A dashed line corresponds to an indirect relationship, while a solid line represents a direct relationship. A. Interaction networks formed by genes regulated by cytoplasmic DDIT3. B. Interaction networks formed by genes regulated by nuclear DDIT3 after 2 hours of tamoxifen treatment. *EGR1* is indicated as a possible network hub (node connecting several paths in the network). C. Interaction networks formed by genes regulated by nuclear DDIT3 after 8 hours of tamoxifen treatment. *EGR1* is indicated as a possible network hub.
(TIF)

Figure S6 **Comparison of regulated genes.** Expression within the morEGFP cell line, within the DDIT3morEGFP cell line, and between both cell lines for genes regulated by cytoplasmic DDIT3. Please note that genes showing differential expression in the morEGFP cell line were excluded in the analysis of nuclear DDIT3 regulation.
(TIF)

Figure S7 **No general DDIT3 effects on cAMP regulated genes.** Log2 fold-changes for 6 genes following treatment with the cAMP inducing agent forskolin in cells expressing EGFP or nuclear DDIT3- EGFP. The gene expression ration between forskolin treated (18 µM) and control cultured cells are shown. The experiment was performed with 3 biological replicates, and differences in induction between the two cell types were assessed with a two-sample t-test (* P-value<0.05). Error bars indicate standard error of the mean values.
(TIF)

Material and Methods S1 **Migration assay modeling.**
(PDF)

Material and Methods S2 **Permutation test for promoter analysis.**
(PDF)

Movie S1 **HT1080cell expressing DDIT3morEGFP recombinant proteins were treated with tamoxifen and the effects were studied photographing every 20 seconds for one hour.**
(MOV)

Table S1 **Sequence of primers used for QPCR analysis.**
(XLS)

Table S2 **Genes regulated by DDIT3.**
(DOC)

Table S3 **QPCR validation of microarray results.**
(XLS)

Table S4 **Functional categories regulated by DDIT3.**
(XLS)

Table S5 **Promoter analysis of DDIT3 regulated genes.**
(XLS)
