# Supplementary figures and images for: Distinct Cytoplasmic and Nuclear Functions of the Stress Induced Protein DDIT3/CHOP/GADD153
Source: PLoS One. 2012 Apr 9;7(4):e33208. doi: 10.1371/journal.pone.0033208 (PMC3322118; doi:10.1371/journal.pone.0033208)

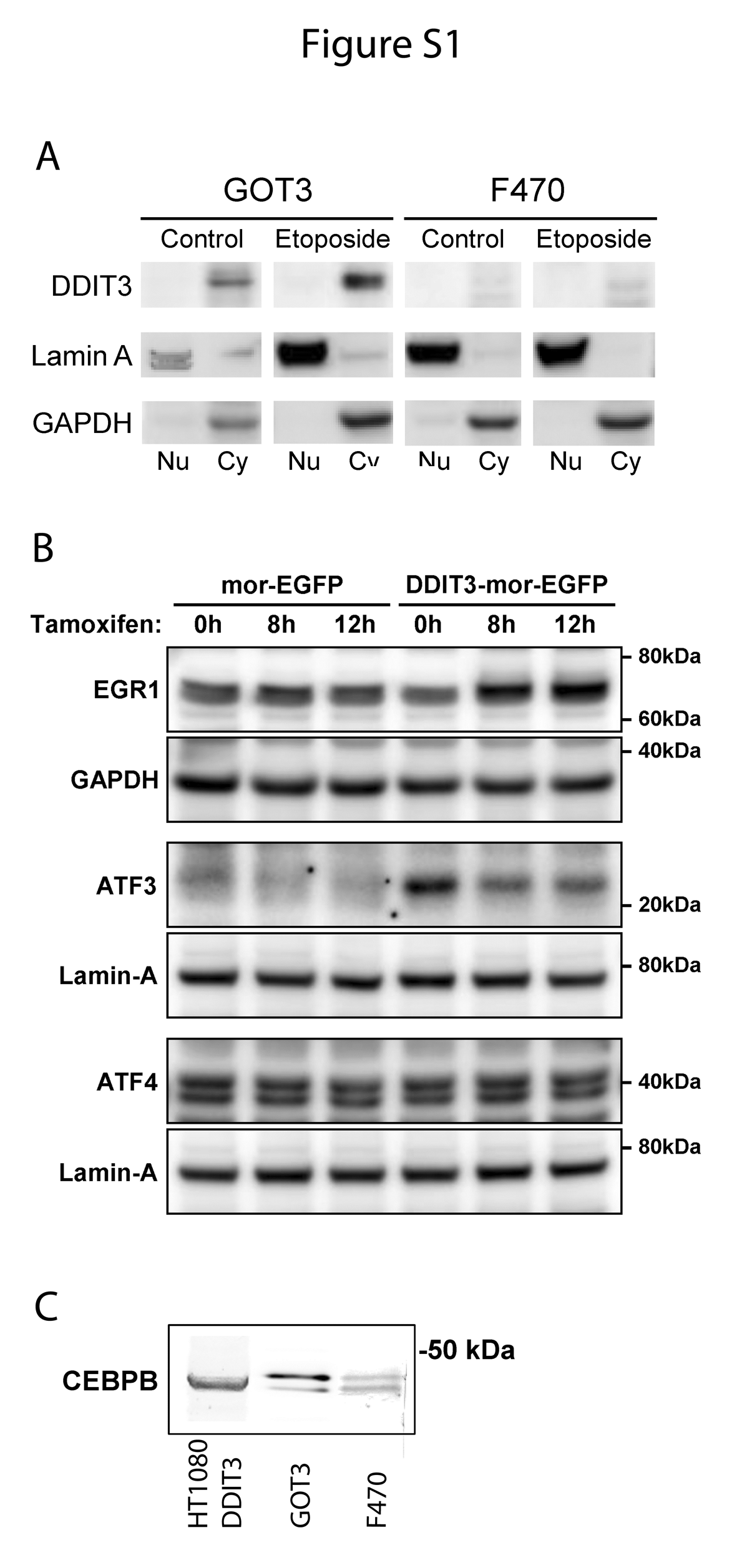

Supplement: Figure S1 — Protein data for GOT3, F470 and HT1080 cells. (A) Etoposide induction of cytoplasmic DDIT3 in human fibroblasts and GOT3 cells. Immunoblot analysis of DDIT3 in nuclear (Nu) and cytoplasmic (Cy) extracts of human liposarcoma cell line GOT3 and normal human fibroblasts F470 following 8 hours of etoposide (30 µM, Etopos) treatment. Cytoplasmic accumulation of DDIT3 is seen in both cell lines compared to untreated cells (Control). GAPDH and Lamin A are cytoplasmic and nuclear markers, respectively. (B) Immunoblot analysis of EGR1, ATF3 and ATF4 in HT1080 cells expressing DDIT3-mor-EGFP or mor-EGFP. Addition of tamoxifen to cell cultures (8 h and 12 h) caused an up-regulation of EGR1 in cells expressing DDIT3-mor-EGFP but not in mor-EGFP control cells. The expression level of ATF3 was increased in cells expressing cytoplasmic DDIT3-mor-EGFP (0 h) as compared to control cells. Nuclear translocation of DDIT3-mor-EGFP (8 h and 12 h) caused a subsequent reduction in ATF3 levels compared to cells with cytoplasmic DDIT3-mor-EGFP. ATF4 was detected in all samples and showed no change in expression levels. GAPDH and Lamin-A were used as loading controls for EGR1 and ATF3/ATF4 respectively. (C) Immunoblot analysis of CEBPB expression in HT1080, GOT3 and cultured human fibroblast (passage 9). (TIF) [file pone.0033208.s001.tif]

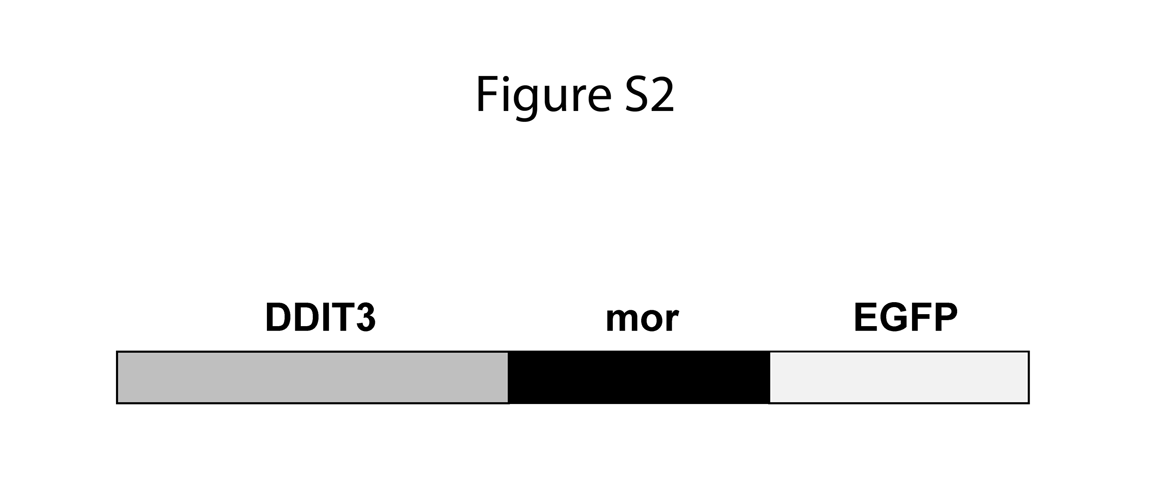

Supplement: Figure S2 — Map of the recombinant DDIT3morEGFP protein. The protein consist of the full length DDIT3 protein juxtaposed to a mutated tamoxifen specific ligand binding part of the mouse estrogen receptor (mor) and EGFP. The mor part mediates cytoplasmic retention that is released upon addition of tamoxifen. (TIF) [file pone.0033208.s002.tif]

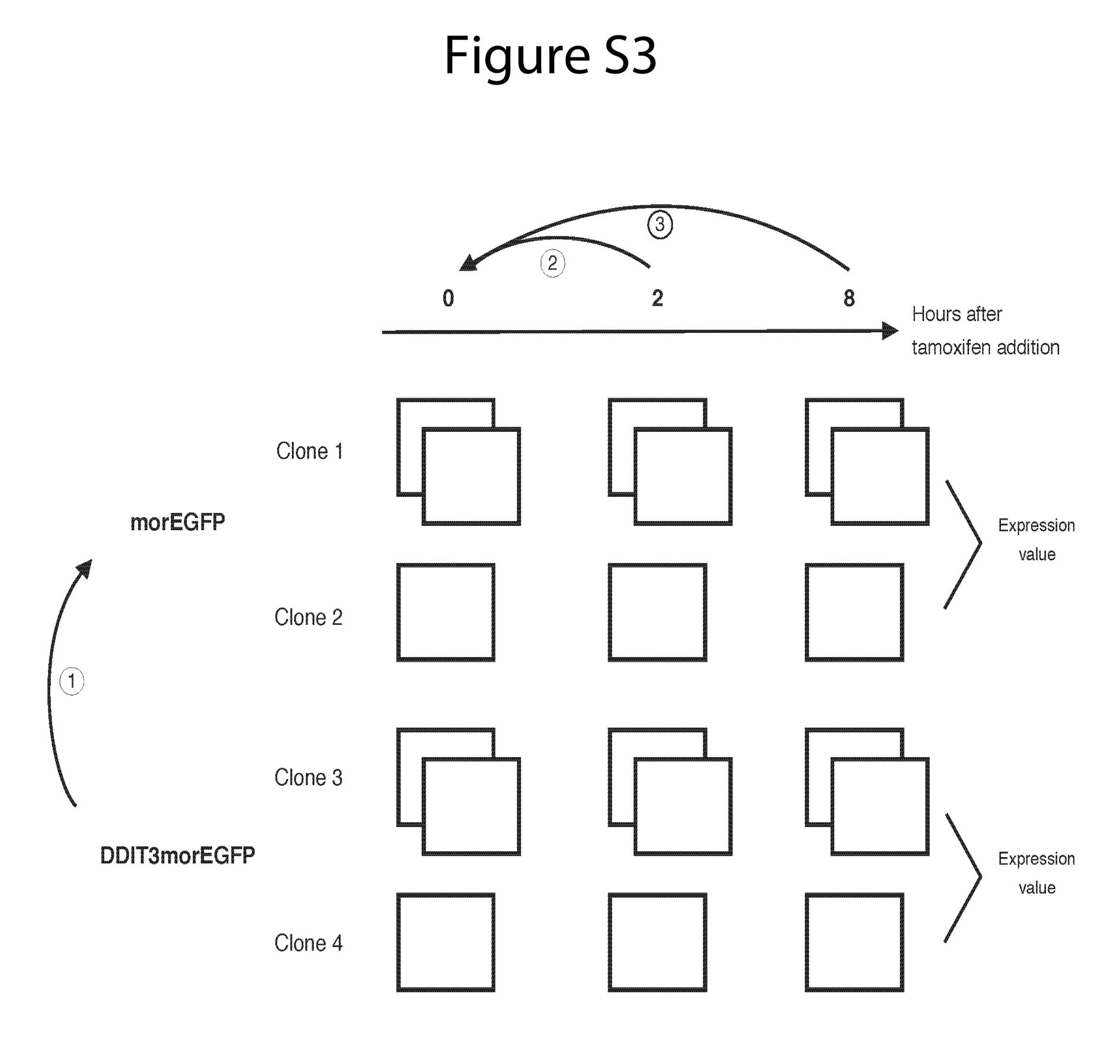

Supplement: Figure S3 — Overview of design and analysis of microarray experiments. Genes regulated by cytoplasmic DDIT3 were extracted by making the comparison indicated with (1), comparing the expression between the morEGFP and DDIT3morEGFP cell lines before addition of tamoxifen. Genes regulated by nuclear DDIT3 were extracted with the comparisons indicated by (2) and (3) within the DDIT3morEGFP cell line, for 2 and 8 hours after tamoxifen addition, respectively. Genes regulated in the morEGFP cell line were removed before analysis of nuclear DDIT3 regulation. Each square corresponds to a microarray experiment replicate. (TIF) [file pone.0033208.s003.tif]

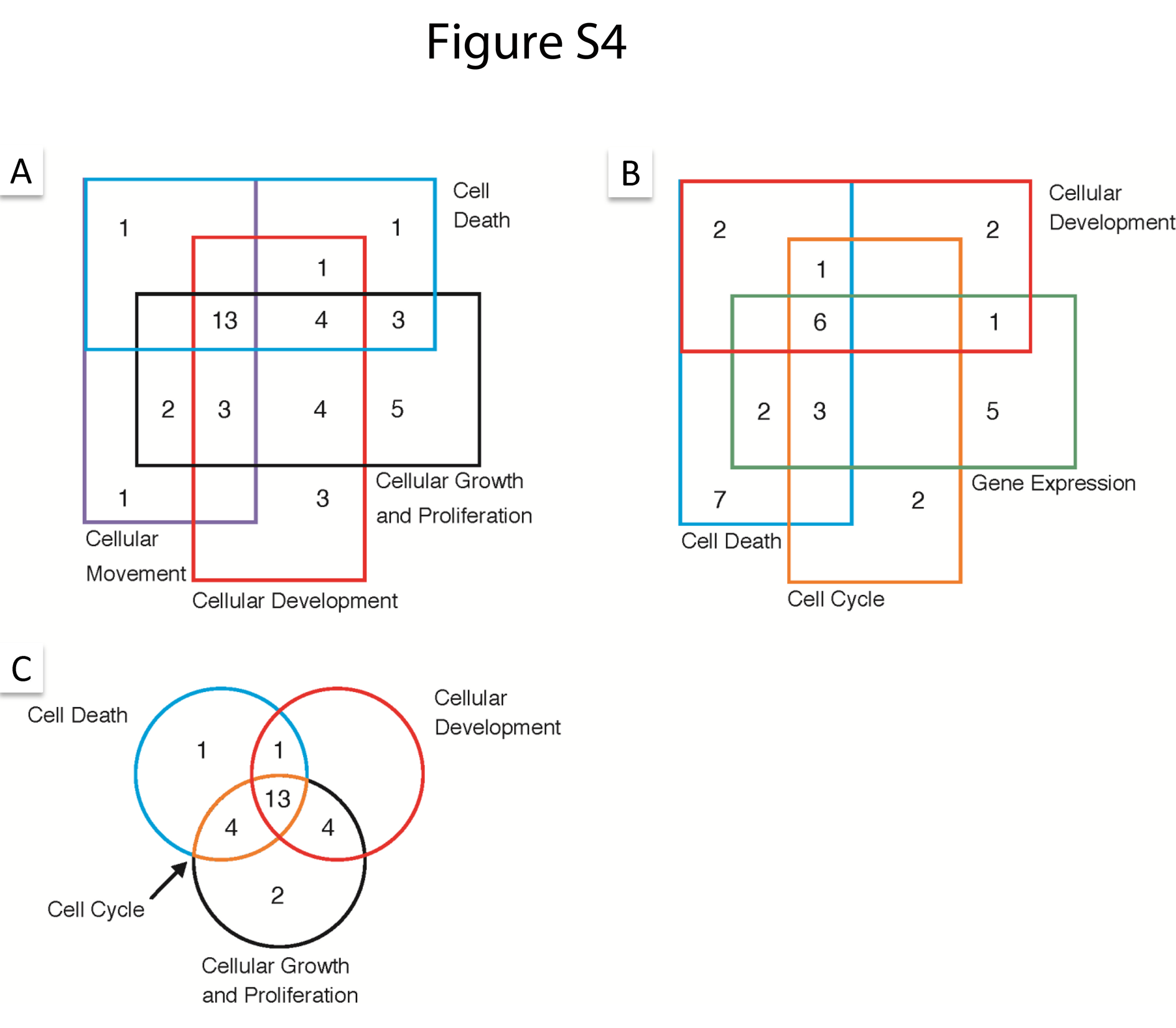

Supplement: Figure S4 — Overlap of genes between functional categories. (a) Cytoplasmic DDIT3 induced genes.(b) Nuclear DDIT3 induced genes after 2 hours of tamoxifen addition. (c) Nuclear DDIT3 induced genes after 8 hours of tamoxifen addition. Four functional categories of genes are shown in (a) and the number of genes that belongs to different categories is shown with overlapping boxes. For example, one gene belongs to both Cell death and Cellular movement, while four genes belong to Cell death, Cellular development and Cellular Growth and Proliferation. (TIFF) [file pone.0033208.s004.tif]

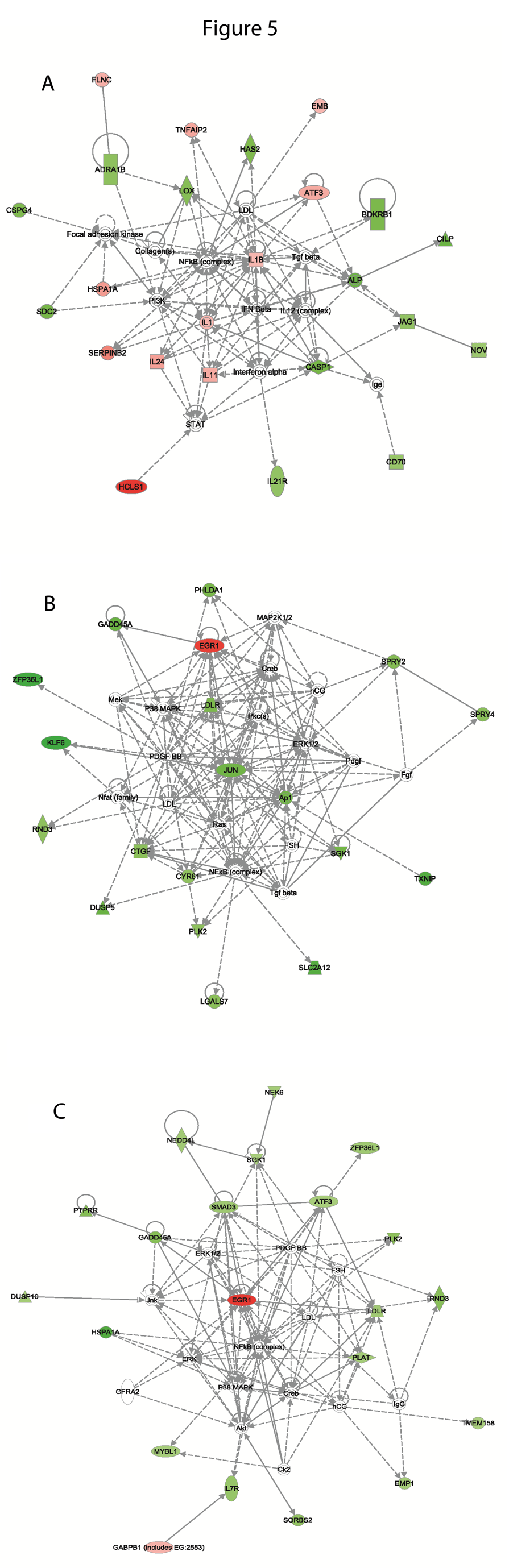

Supplement: Figure S5 — Interaction networks formed by the genes regulated by DDIT3. Red hues correspond to upregulation, while green hues correspond to downregulation (no coloring means no differential expression). Vertical diamonds, horizontal diamonds, vertical ovals, and horizontal ovals represent enzymes, peptidases, transmembrane receptors, and transcription factors, respectively. Squares, rectangles, up-pointing triangles, and down-pointing triangles denote cytokines, G-protein coupled receptors, phosphatases, and kinases, in turn. Double circles, trapezoids, and single circles symbolize complexes, transporters, and finally, gene products with other functions. A dashed line corresponds to an indirect relationship, while a solid line represents a direct relationship. A. Interaction networks formed by genes regulated by cytoplasmic DDIT3. B. Interaction networks formed by genes regulated by nuclear DDIT3 after 2 hours of tamoxifen treatment. EGR1 is indicated as a possible network hub (node connecting several paths in the network). C. Interaction networks formed by genes regulated by nuclear DDIT3 after 8 hours of tamoxifen treatment. EGR1 is indicated as a possible network hub. (TIF) [file pone.0033208.s005.tif]

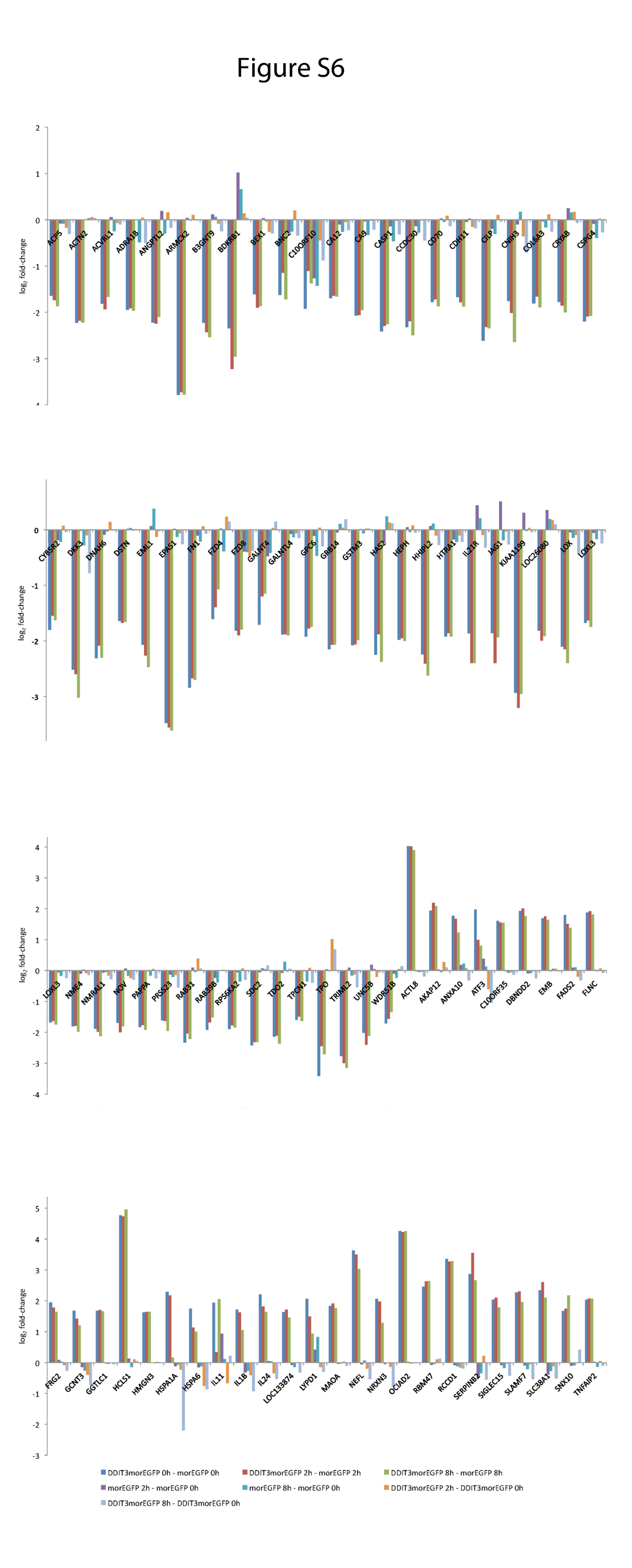

Supplement: Figure S6 — Comparison of regulated genes. Expression within the morEGFP cell line, within the DDIT3morEGFP cell line, and between both cell lines for genes regulated by cytoplasmic DDIT3. Please note that genes showing differential expression in the morEGFP cell line were excluded in the analysis of nuclear DDIT3 regulation. (TIF) [file pone.0033208.s006.tif]

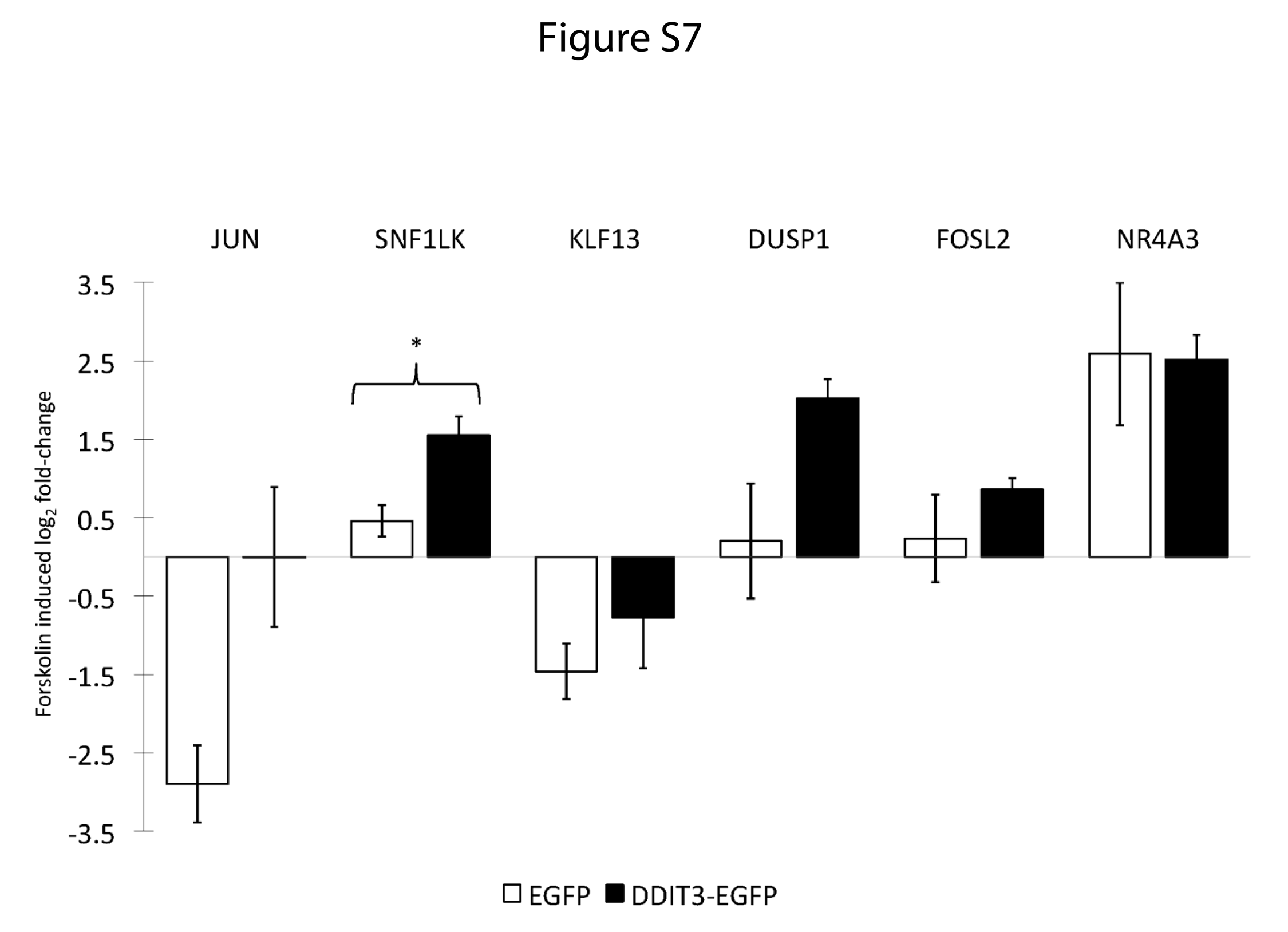

Supplement: Figure S7 — No general DDIT3 effects on cAMP regulated genes. Log2 fold-changes for 6 genes following treatment with the cAMP inducing agent forskolin in cells expressing EGFP or nuclear DDIT3- EGFP. The gene expression ration between forskolin treated (18 µM) and control cultured cells are shown. The experiment was performed with 3 biological replicates, and differences in induction between the two cell types were assessed with a two-sample t-test (* P-value<0.05). Error bars indicate standard error of the mean values. (TIF) [file pone.0033208.s007.tif]
