# Supplementary material for: Distinct Cytoplasmic and Nuclear Functions of the Stress Induced Protein DDIT3/CHOP/GADD153
Source: PLoS One. 2012 Apr 9;7(4):e33208. doi: 10.1371/journal.pone.0033208 (PMC3322118; doi:10.1371/journal.pone.0033208)
Supplement: Material and Methods S1 — Migration assay modeling. (PDF) [file pone.0033208.s008.pdf]

Distinct cytoplasmic and nuclear functions of the stress induced protein DDIT3/CHOP/GADD153

Alexandra Jauhiainen, Christer Thomsen, Linda Strömbom, Pernilla Grundevik, Carola Andersson, Anna Danielsson, Mattias K Andersson, Olle Nerman, Linda Rörkvist, Anders Ståhlberg and Pierre Åman.

Supporting Materials and Methods S1

Cell migration assay modeling

The experimental setup for the migration assay is described in the main text. Initially, six replicates for three separate experiments on the migration rates for EGFP, DDIT3-EGFP, DDIT3morEGFP were made. To test the effect of tamoxifen on the migration rate, an additional four experiments on the migration rate for EGFP and DDIT3morEGFP were produced (with and without tamoxifen). The assay employed differs from traditional scratch wound assays by the use of two cell types that are seeded and co-migrate together. The use of wild type cells as reference in the experiments decreases experimental variability and possible contributions of cell proliferation to the migration assay differences.

To model the migration rates (probabilities) for two different cell types, for example EGFP and DDIT3-EGFP, we make the following assumptions. Let the different proportions of EGFP-stained cells to wild type cells in the two experiments to be compared be denoted by $\pi = (\pi_1, \pi_2)$ and let M denote the event that a cell has migrated into the scratch wound. From each experiment i we can estimate the conditional probability that a cell is green (stained with EGFP) given that it has migrated: $p^{(i)}_{G|M} = P(G^{(i)}|M)$ simply as the proportion of green cells in the scratch(es). What we wish to investigate is the migration rate for the EGFP-stained cells in each experiment, i.e. the probabilities $P(M|G^{(i)})$. We denote the migration probability for wild type cells with p and the migration probability for transfected cells with $p + \delta^{(i)}$ in experiment i. We can now apply Bayes theorem.

$$\begin{aligned} p^{(i)}_{G|M} = P(G^{(i)}|M) &= \frac{P(M|G^{(i)})P(G^{(i)})}{P(M|G^{(i)})P(G(i)) + P(M|W^{(i)})P(W^{(i)})} \\ &= \frac{(p+\delta^{(i)})\pi_i}{(p+\delta^{(i)})\pi_i + p(1-\pi_i)} \end{aligned} \tag{1}$$

where W denotes wild type cells and G transfected (EGFP-stained) cells. With some algebra we can by rearranging the terms in the above equality deduce

$$\frac{p}{p+\delta^{(i)}} = \frac{\pi_i(1-p^{(i)}_{G|M})}{(1-\pi_i)p^{(i)}_{G|M}}. \tag{2}$$

We estimate this quantity for all replications by plugging in the estimates for π_i and $p^{(i)}_{G|M}$.

To compare the migration rates for the two chosen cell types, we use all pairwise quotients of the ratios deduced in the above formula.

$$\frac{\widehat{p+\delta^{(2)}}}{p+\delta^{(1)}} = \frac{\hat{\pi}_1(1-\hat{p}^{(1)}_{G|M})}{(1-\hat{\pi}_1)\hat{p}^{(1)}_{G|M}} \Bigg/ \frac{\hat{\pi}_2(1-\hat{p}^{(2)}_{G|M})}{(1-\hat{\pi}_2)\hat{p}^{(2)}_{G|M}} \tag{3}$$

By noting how many of the quotients are larger than one, we get the Mann-Whitney U statistic and we can use the Mann-Whitney U test (equivalent to a Wilcoxon rank sum test)[1]. The hypotheses we test are whether observations from one population exceed the observations from another population or not, i.e. if the migration probability in one group exceeds the migration probability in another group.

Migration assay experiments and results

The first set of experiments was designed to test differences in migration rates without the addition of tamoxifen. Cell lines transfected with EGFP, DDIT3-EGFP, and DDIT3morEGFP were seeded to petri dishes with wild type cells in separate experiments. Six replicates were made for each transfected cell line and cells counted after migration into the scratch wound. Background proportions (i.e. the π_i's) of transfected cells were calculated by counting cells in non-wounded areas, and each proportion was pooled to a global estimate for each transfected cell type. The data is given below in Tables 1-2.

From this set of experiments quotients, as given in Equation (3) above, were estimated for three comparisons (36 quotients in each). The quotients of ratios between EGFP and DDIT3-EFGP, likewise for EGFP and DDIT3morEGFP, deviate from one in the same direction. This corresponds to the most extreme outcome of the statistic and gives a p-value of approximately 0.002. For the ratio between DDIT3morEFGP and DDIT3-EGFP migration probabilities, we observe a p-value of 0.065. We can hence deduce that the migration probabilities between DDIT3morEFGP and DDIT3-EGFP most likely differ from the migration rate of EGFP cells, but we cannot on the 0.05 level claim that the migration probabilities are different in the two DDIT3 groups.

The second set of experiments was created to also test the effect of tamoxifen on migration (tamoxifen concentration the same as in the other experiments, 100 nM). Cell lines transfected with morEGFP and DDIT3morEGFP were used together with wildtype cells. 10 replicates were made of each experiment with morEGFP and DDIT3morEGFP without tamoxifen. Cells were counted in the scratch wound and in non-wounded areas for all replicates, which resulted in background proportions (i.e. the π_i's) estimated individually for each assay (not pooled to a global estimate as in the first set). In the experiments with tamoxifen, 11 replicates were made for morEGFP and DDIT3morEGFP, and cells counted in both scratch wound and non-wounded areas. The data is given below in Tables 3-4.

The fact that the background proportions were measured individually gives independence between the estimated ratios which better fulfills the assumptions in the Mann-Whitney U test.

The follow-up experiment confirmed the difference in migration rate between DDIT3morEFGP and morEGFP without tamoxifen (p-value of 0.004). Also, tamoxifen did not seem to affect the migration rates since a difference in rates between DDIT3morEFGP with tamoxifen and DDIT3morEFGP without tamoxifen (p-value 0.65) as well as a difference between EGFP with and without tamoxifen (p-value 0.31) could not be shown. A difference in migration rate between DDIT3morEFGP and EGFP with tamoxifen could also be shown (p-value 0.005).

[1]**Agresti, A**. 2002. Categorical Data Analysis, 2nd ed. Wiley-Interscience, New York, NY.

EGFP		DDIT3-EGFP		DDIT3morEGFP	
(R)	(G)	(R)	(G)	(R)	(G)
125	82	71	12	95	35
109	69	79	12	144	57
118	74	67	21	161	65
168	107	72	28	115	31
131	76	84	23	126	45
134	91	76	18	98	29

Table 1: Scratch wound assay cell counts in the first set of experiments. Numbers in columns named (R) represent all cells that have migrated into a wound (red), while number in columns with heading (G) represent all transfected cells (green). Note that no tamoxifen treated cells were included in this set.

π_{EGFP}	$\pi_{\mathrm{DDIT3-EGFP}}$	$\pi_{\mathrm{DDIT3morEGFP}}$
0.512	0.476	0.470

Table 2: Estimated background proportions (π) of transfected cells in the first set of experiments. The counts were observed in non-wounded areas of the assays and pooled to one estimate per transfected cell line.

Without tamoxifen				With tamoxifen			
morEGFP		DDIT3morEGFP		morEGFP		DDIT3morEGFP	
(R)	(G)	(R)	(G)	(R)	(G)	(R)	(G)
173	96	484	190	304	137	329	96
88	46	391	123	204	109	260	72
165	90	422	114	307	172	135	39
251	110	384	120	441	200	335	53
300	199	502	208	232	153	196	36
252	130	386	188	167	75	92	23
124	70	124	41	412	174	85	18
152	75	158	75	110	60	100	26
178	91	163	49	188	98	153	39
136	72	166	26	208	126	130	42
				142	90	185	51

Table 3: Scratch wound assay cell counts in the second set of experiments. Numbers in columns named (R) represent all cells that have migrated into a wound (red), while number in columns with heading (G) represent all transfected cells (green).

Without tamoxifen		With tamoxifen	
π_{morEGFP}	$\pi_{\text{DDIT3morEGFP}}$	π_{morEGFP}	$\pi_{\text{DDIT3morEGFP}}$
0.571	0.492	0.48	0.421
0.446	0.491	0.45	0.436
0.46	0.464	0.587	0.376
0.56	0.452	0.59	0.415
0.5	0.522	0.607	0.381
0.491	0.533	0.491	0.381
0.472	0.538	0.529	0.388
0.561	0.449	0.535	0.328
0.548	0.417	0.641	0.22
0.648	0.515	0.444	0.474
		0.769	0.524

Table 4: Estimated background proportions (π) of transfected cells in the second set of experiments. The counts were observed in non-wounded areas of the assays.
