# Supplementary material for: Distinct Cytoplasmic and Nuclear Functions of the Stress Induced Protein DDIT3/CHOP/GADD153
Source: PLoS One. 2012 Apr 9;7(4):e33208. doi: 10.1371/journal.pone.0033208 (PMC3322118; doi:10.1371/journal.pone.0033208)
Supplement: Material and Methods S2 — Permutation test for promoter analysis. (PDF) [file pone.0033208.s009.pdf]

Distinct cytoplasmic and nuclear functions of the stress induced protein DDIT3/CHOP/GADD153

Alexandra Jauhiainen, Christer Thomsen, Linda Strömbom, Pernilla Grundevik, Carola Andersson, Anna Danielsson, Mattias K Andersson, Olle Nerman, Linda Rörkvist, Anders Ståhlberg and Pierre Åman.

Supporting Materials and Methods S2

Permutation test for TFBS enrichment

The test to detect enrichment for TFBS among the regulated genes was based on a weighted statistic and significance assessed with permutation. We assume that we have expression values for a set of genes in two conditions. The genes are ranked for differential expression using for example $\log_2$-fold change, or the moderated t-statstic. These gene level statistics are denoted by d_g. We also have a set of scores for the occurrence of motifs in the promoter of each gene. The indicator I_{gj} equals 1 if gene g contains motif j in its promoter and 0 otherwise. We use the following test statistic

$$u = \sum_g w_{gj}(d_g) I_{gj}$$

with weights w_{gj}. The weights score the values of the gene level between 0 and 1. If a gene is highly differentially expressed, it receives a score close to 1, otherwise it should receive a score close to 0. We use a logistic curve for the weights, for which we can vary the location and scale parameters according to the gene expression data.

If a motif is present in the promoter of several differentially expressed genes, the weights will be closer to 1 for these genes, and the test statistic u should be large. Conversely, if a motif is rarely seen in the promoters of the differentially expressed genes, it results in a small value of u.

The significance of motif occurrence and high differential expression is tested with permutation on the indicators I_{gj}. The motif occurrence is permuted 1000 times and the value of the test statistic u is calculated for each permutation. The p-value for enrichment of motif j among the differentially expressed genes is calculated as

$$P = \sum_p I(u_p > u)p$$

where up denotes the value of the statistic in permutation p.

The parameter values for the location and scale parameters in the weigh functions have to be chosen by the user, but we recommend setting the location parameter to roughly the 80%-quantile of the gene level statistics.

We compared our method with another common permutation procedure called Gene Set En-

richment Analysis (GSEA)[1] using a simulation study previously described[2]. Briefly, the expression for 600 genes in 20 samples was simulated using a multivariate normal distribution (all with variance 1). 520 genes constituted the background set, and were simulated with a mean $\mu = 0$ and correlation $\rho = 0$. The remaining 80 genes were simulated with different means and correlations mixed of values $\mu = (0.75, 1, -1)$ and $\rho = (0, 0.6, -0.6)$. Nine sets were used to test the enrichment methods, of which sets 1, 2, 6, and 7 should be detected by any well working method, and sets 4, 5, 8, 9 ideally also should be detected (although only half of the genes were differentially expressed in these sets). Set 3 should work as a negative control[2].

We simulated 100 data sets, ranked the genes in each data set by $\log_2$-fold change (absolute values) as well as by the moderate-t statistic (also absolute values), and tested each method on these sets. Our method was tested with three different values on the location parameter, corresponding to the 75, 80, and 85 percentiles of the gene level statistics. The scale parameter was set to 0.1.

We observe that the results for the permutation test performs slightly better than GSEA on all data sets. The results seem to be quite robust to the choice of the location parameter. The scale parameter can also be varied, an influences how sharply the logistic curve switches from values close to zero to values close to one. The results are quite robust also to the choice of this parameter (data not shown), but we recommend values in the range 0.05 - 0.2. According to our simulations, a good choice for the location parameter is in the range given above (75-85th percentiles of the gene level statistics).

Our permutation method is very easy to implement and the better performance of our statistic u to the running sum in the GSEA is probably due to the fact that our statistic is not sensitive in the same way to the absolute gene ranking. Although there is a need to choose the extra location and scale parameters, our method offers more versatility in how the expression values are allowed to influence the results (we can choose to only use highly differentially expressed genes, or be more liberal and allow genes with moderate expression values to also influence the statistic). We can choose to apply the weights (the logistic curve) to absolute values of the gene level statistics, or to the original values.

For the motif enrichment p-values presented in the paper (see Table S4 in supplemental material), we ranked the genes by absolute $\log_2$-fold change and chose the values 0.75 for the location parameter and 0.1 for the scale parameter. We also tested the method on the down regulated genes, with similar negative results (data not shown).

[1] **Subramanian, A. et al.** 2005. Gene set enrichment analysis: a knowledge-based approach for interpreting genome-wide expression profiles. Proc Natl Acad Sci U S A. **102**(43):15545-50.

[2] **Ackerman, M. and K. Strimmer.** 2009. A general modular framework for gene set enrichment ananlysis. BMC Bioinformatics. **10**:47.

	$\log_2$-fold change	moderated-t
set 1	0.62	0.63
set 2	0.93	0.93
set 3	0	0
set 4	0.47	0.47
set 5	0.45	0.43
set 6	0.89	0.89
set 7	1	1
set 8	0.71	0.73
set 9	0.85	0.84

Table 1: Enrichment results from GSEA. The values correspond to the proportion of p-values < 0.05 in the 100 data sets.

	$\log_2$-fold change (1)	$\log_2$-fold change (2)	$\log_2$-fold change (3)	moderated-t (1)	moderated-t (2)	moderated-t (3)
set 1	0.7	0.67	0.64	0.7	0.66	0.63
set 2	0.99	0.99	0.99	0.99	0.99	0.98
set 3	0	0	0	0	0	0
set 4	0.56	0.55	0.51	0.57	0.52	0.5
set 5	0.52	0.56	0.55	0.52	0.52	0.49
set 6	0.92	0.92	0.88	0.92	0.9	0.86
set 7	1	1	1	1	1	1
set 8	0.78	0.78	0.76	0.77	0.75	0.75
set 9	0.89	0.96	0.95	0.86	0.93	0.9

Table 2: Enrichment results from our permutation test. The values correspond to the proportion of p-values < 0.05 in the 100 data sets. The location parameter was set to 0.6, 0.68, and 0.77 for the $\log_2$-fold change ranked data and to 1.35, 1.52, and 1.73 for the data ranked with the moderated-t statistic. The scale parameter was set to 0.1
