# Supplementary material for: Distinct Cytoplasmic and Nuclear Functions of the Stress Induced Protein DDIT3/CHOP/GADD153
Source: PLoS One. 2012 Apr 9;7(4):e33208. doi: 10.1371/journal.pone.0033208 (PMC3322118; doi:10.1371/journal.pone.0033208)
Supplement: Table S2 — Genes regulated by DDIT3. (DOC) [file pone.0033208.s012.doc]

**Table S2: Genes regulated by DDIT3.**

| **Gene** | **Nuclear 2h** | **Nuclear 8h** | **Cytoplasmic** | **Functional Category** |
| --- | --- | --- | --- | --- |
| *FAM189A2* | 1.29 |  |  |  |
| *HBB* | 1.06 |  |  | Cell Dev |
| *KIAA1609* | 1.03 |  |  |  |
| *TPO** | 1.02 |  | -3.42 | Cell Growth |
| *ZNF30* | 1.21 |  |  |  |
| *ARHGAP12* | -1.01 |  |  |  |
| *CDC42EP2* | -1.1 |  |  |  |
| *CLEC14A* | -1.12 |  |  |  |
| *CTGF* | -1.05 |  |  | Cell Death, Cell Dev, Cell Growth |
| *CTNNA1* | -1.04 |  |  | Cell Death, Cell Dev, Cell Growth |
| *CYR61* | -1.01 |  |  | Cell Death, Cell Dev, Cell Growth, Cell Cycle |
| *DTWD2* | -1.52 |  |  |  |
| *DUSP5* | -1.4 |  |  | Cell Death, Cell Dev, Cell Growth |
| *GP1BA* | -1.09 |  |  | Cell Dev, Cell Growth |
| *HOOK1* | -1.28 |  |  | Cell Dev |
| *IL20* | -1.4 |  |  | Cell Dev, Cell Growth |
| *JUN** | -1.3 |  |  | Cell Death, Cell Dev, Cell Growth, Cell Cycle |
| *KLF6* | -1.81 |  |  | Cell Death, Cell Growth, Cell Cycle |
| *LGALS7* | -1.05 |  |  | Cell Death, Cell Dev, Cell Growth |
| *LHFPL1* | -1.19 |  |  |  |
| *LOC285696* | -1.02 |  |  |  |
| *PAX2* | -1.53 |  |  | Cell Death, Cell Dev, Cell Growth |
| *PDXDC1* | -1.09 |  |  |  |
| *PHLDA1* | -1.24 |  |  | Cell Death, Cell Growth |
| *PRNT* | -1.38 |  |  |  |
| *SLC2A12* | -1.59 |  |  |  |
| *SOX9* | -1.03 |  |  | Cell Death, Cell Dev, Cell Growth, Cell Cycle |
| *SPRY2* | -1.11 |  |  | Cell Death, Cell Dev, Cell Growth, Cell Cycle |
| *SPRY4* | -1.06 |  |  | Cell Dev, Cell Growth |
| *SYVN1* | -1.98 |  |  | Cell Death |
| *TMEM95* | -1.82 |  |  |  |
| *UBQLN3* | -1.63 |  |  |  |
| *ALDH16A1* | 1.03 | 1.59 |  |  |
| *ATF7IP* | 1.03 | 1.18 |  |  |
| *CSRNP2* | 1.35 | 1.3 |  |  |
| *EGR1** | 1.23 | 3.42 |  | Cell Death, Cell Dev, Cell Growth, Cell Cycle |
| *GTPBP4* | 1.27 | 1.42 |  | Cell Dev, Cell Growth |
| *GADD45A** | -1.4 | -1.46 |  | Cell Death, Cell Dev, Cell Growth, Cell Cycle |
| *IRF2BP2* | -1.08 | -1.18 |  |  |
| *LDLR* | -1.21 | -1.12 |  | Cell Death, Cell Growth |
| *PLK2** | -1.02 | -1.39 |  | Cell Death, Cell Dev, Cell Growth, Cell Cycle |
| *RND3** | -1.03 | -1.43 |  | Cell Death, Cell Dev |
| *SGK1** | -1.28 | -1.04 |  | Cell Death, Cell Growth, Cell Cycle |
| *TXNIP* | -1.62 | -1.61 |  | Cell Death, Cell Dev, Cell Growth, Cell Cycle |
| *ZFP36L1* | -1.78 | -1.16 |  | Cell Growth |
| *AMMECR1L* |  | 1.11 |  |  |
| *BCORL1* |  | 1.36 |  |  |
| *C12ORF34* |  | 1.14 |  |  |
| *C3ORF39* |  | 1.67 |  |  |
| *CYB5R1* |  | 1.18 |  |  |
| *GABPB1* |  | 1.29 |  | Cell Death |
| *KIAA0528* |  | 1.1 |  |  |
| *KIF23** |  | 1.14 |  | Cell Cycle, Cell Growth |
| *MAPRE1** |  | 1.13 |  | Cell Dev, Cell Growth |
| *RAD54L2* |  | 1.07 |  |  |
| *RBM45* |  | 1.08 |  |  |
| *SNAPIN* |  | 1.09 |  |  |
| *TTC5* |  | 1.12 |  | Cell Death |
| *VPS54* |  | 1.07 |  |  |
| *AGPAT9* |  | -1.35 |  |  |
| *ATF3** |  | -1.05 | 1.98 | Cell Death, Cell Dev, Cell Cycle, Cell Growth, Cell Move |
| *DENND3* |  | -1.16 |  |  |
| *DUSP10* |  | -1.11 |  | Cell Death, Cell Growth |
| *EMP1* |  | -1.09 |  | Cell Death, Cell Growth |
| *HIVEP2* |  | -1.33 |  | Cell Dev |
| *HSPA1A* |  | -2.2 | 2.29 | Cell Death, Cell Cycle, Cell Growth |
| *IL7R** |  | -1.25 |  | Cell Death, Cell Dev, Cell Growth |
| *MCHR2* |  | -2.65 |  |  |
| *MYBL1* |  | -1.06 |  | Cell Death, Cell Dev, Cell Cycle, Cell Growth |
| *NEDD4L* |  | -1.14 |  | Cell Cycle |
| *NEK6* |  | -1.1 |  | Cell Death, Cell Cycle |
| *PDP1* |  | -1.19 |  | Cell Death |
| *PLAT* |  | -1.08 |  | Cell Death, Cell Dev, Cell Growth |
| *PTPRR* |  | -1.49 |  | Cell Growth |
| *S1PR4* |  | -1.01 |  |  |
| *SH3D20* |  | -1.16 |  |  |
| *SH3PXD2A* |  | -1.08 |  |  |
| *SMAD3** |  | -1.16 |  | Cell Death, Cell Dev, Cell Cycle, Cell Growth |
| *SORBS2* |  | -1.32 |  | Cell Death |
| *SYNGR2* |  | -1.07 |  |  |
| *TCEAL1* |  | -1.13 |  |  |
| *TMEM158* |  | -1.03 |  | Cell Death |
| *TOX2* |  | -1.03 |  |  |
| *TPST1* |  | -1.41 |  |  |
| *HCLS1* |  |  | 4.76 | Cell Death, Cell Growth |
| *OCIAD2* |  |  | 4.25 |  |
| *ACTL8* |  |  | 4.03 |  |
| *NEFL* |  |  | 3.63 | Cell Death |
| *RCCD1* |  |  | 3.36 |  |
| *SERPINB2* |  |  | 2.87 | Cell Move, Cell Death, Cell Dev, Cell Growth |
| *RBM47* |  |  | 2.46 |  |
| *SLC38A1* |  |  | 2.34 |  |
| *SLAMF7* |  |  | 2.28 | Cell Growth |
| *IL24* |  |  | 2.21 | Cell Death, Cell Dev, Cell Growth |
| *LYPD1* |  |  | 2.06 |  |
| *NRXN3* |  |  | 2.06 |  |
| *SIGLEC15* |  |  | 2.04 |  |
| *TNFAIP2* |  |  | 2.04 |  |
| *FRG2* |  |  | 1.95 |  |
| *IL11* |  |  | 1.94 | Cell Move, Cell Death, Cell Dev, Cell Growth |
| *AKAP12* |  |  | 1.94 | Cell Death, Cell Dev, Cell Growth |
| *DBNDD2* |  |  | 1.93 |  |
| *FLNC* |  |  | 1.88 | Cell Dev |
| *MAOA* |  |  | 1.84 | Cell Move, Cell Death |
| *FADS2* |  |  | 1.8 | Cell Growth |
| *ANXA10* |  |  | 1.77 |  |
| *HSPA6* |  |  | 1.74 |  |
| *IL1B* |  |  | 1.71 | Cell Move, Cell Death, Cell Dev, Cell Growth |
| *EMB* |  |  | 1.7 |  |
| *GCNT3* |  |  | 1.67 |  |
| *GGTLC1* |  |  | 1.67 |  |
| *SNX10* |  |  | 1.67 |  |
| *LOC133874* |  |  | 1.64 |  |
| *HMGN3* |  |  | 1.63 |  |
| *C10ORF35* |  |  | 1.61 |  |
| *ARMCX2* |  |  | -3.79 |  |
| *EPAS1* |  |  | -3.48 | Cell Dev, Cell Growth |
| *KIAA1199* |  |  | -2.93 |  |
| *FN1* |  |  | -2.84 | Cell Move, Cell Death, Cell Dev, Cell Growth |
| *TRIML2* |  |  | -2.77 |  |
| *CILP* |  |  | -2.61 |  |
| *DKK3* |  |  | -2.52 | Cell Move, Cell Death, Cell Dev, Cell Growth |
| *SDC2* |  |  | -2.42 | Cell Move, Cell Death, Cell Dev, Cell Growth |
| *CASP1* |  |  | -2.41 | Cell Move, Cell Death, Cell Dev, Cell Growth |
| *BDKRB1* |  |  | -2.34 | Cell Move, Cell Death, Cell Dev, Cell Growth |
| *RAB31* |  |  | -2.33 |  |
| *CCDC30* |  |  | -2.32 |  |
| *DNAH6* |  |  | -2.31 |  |
| *HAS2* |  |  | -2.25 | Cell Move, Cell Death, Cell Dev, Cell Growth |
| *HHIPL2* |  |  | -2.24 |  |
| *B3GNT9* |  |  | -2.23 |  |
| *ACTN2* |  |  | -2.23 | Cell Dev |
| *ANGPTL2* |  |  | -2.22 | Cell Dev |
| *CSPG4* |  |  | -2.2 | Cell Move, Cell Death, Cell Dev, Cell Growth |
| *GRB14* |  |  | -2.15 | Cell Dev, Cell Growth |
| *TDO2* |  |  | -2.14 |  |
| *LOX* |  |  | -2.1 | Cell Move, Cell Dev, Cell Growth |
| *GSTM3* |  |  | -2.08 |  |
| *CA9* |  |  | -2.07 | Cell Death, Cell Dev, Cell Growth |
| *EML1* |  |  | -2.07 |  |
| *UNC5B* |  |  | -2.02 | Cell Move, Cell Death, Cell Dev, Cell Growth |
| *HEPH* |  |  | -1.98 |  |
| *ADRA1B* |  |  | -1.95 | Cell Growth |
| *HTRA1* |  |  | -1.92 | Cell Growth |
| *C10ORF10* |  |  | -1.92 |  |
| *GPC6* |  |  | -1.92 |  |
| *RAB39B* |  |  | -1.92 |  |
| *RPS6KA2* |  |  | -1.9 | Cell Death, Cell Dev |
| *NMRAL1* |  |  | -1.89 |  |
| *GALNTL4* |  |  | -1.88 |  |
| *IL21R* |  |  | -1.87 | Cell Dev, Cell Growth |
| *JAG1* |  |  | -1.86 | Cell Move, Cell Death, Cell Dev, Cell Growth |
| *PAPPA* |  |  | -1.83 | Cell Move, Cell Growth |
| *FZD8* |  |  | -1.82 |  |
| *LOC26080* |  |  | -1.81 |  |
| *ACVRL1* |  |  | -1.81 | Cell Move, Cell Dev, Cell Growth |
| *COL6A3* |  |  | -1.81 | Cell Dev, Cell Growth |
| *NME4* |  |  | -1.81 |  |
| *CYB5R2* |  |  | -1.8 |  |
| *CD70* |  |  | -1.78 | Cell Death, Cell Dev, Cell Growth |
| *CRYAB* |  |  | -1.78 | Cell Death, Cell Growth |
| *CNIH3* |  |  | -1.76 |  |
| *WDR51B* |  |  | -1.71 |  |
| *GALNT4* |  |  | -1.71 |  |
| *CA12* |  |  | -1.7 |  |
| *NOV* |  |  | -1.69 | Cell Move, Cell Dev, Cell Growth |
| *LOXL3* |  |  | -1.67 |  |
| *CDH11* |  |  | -1.67 | Cell Move, Cell Growth |
| *ACP5* |  |  | -1.64 |  |
| *DSTN* |  |  | -1.64 | Cell Move |
| *BNC2* |  |  | -1.63 |  |
| *PRSS23* |  |  | -1.61 |  |
| *FZD4* |  |  | -1.61 |  |
| *BEX1* |  |  | -1.61 |  |
| *TPCN1* |  |  | -1.59 |  |

Differentially expressed genes (log2 fold-change) for cytoplasmic and nuclear DDIT3. The functional categories are abbreviated as: Cell Move : Cellular Movement, Cell Dev: Cellular Development, Cell Growth: Cellular Growth and Proliferation. The expression for genes marked by an asterisk (*) has been validated by PCR (Table S2). Complete lists of functional categories are shown in Table S3A-C.
